# Calcium Orthophosphates as Bioceramics: State of the Art

**DOI:** 10.3390/jfb1010022

**Published:** 2010-11-30

**Authors:** Sergey V. Dorozhkin

**Affiliations:** Kudrinskaja sq. 1-155, Moscow 123242, Russia; E-Mail: sedorozhkin@yandex.ru; Tel.: +7-499-255-4460

**Keywords:** calcium orthophosphates, hydroxyapatite, bioceramics, biomaterials, biomedical applications, bone grafts, tissue engineering

## Abstract

In the late 1960s, much interest was raised in regard to biomedical applications of various ceramic materials. A little bit later, such materials were named bioceramics. This review is limited to bioceramics prepared from calcium orthophosphates only, which belong to the categories of bioactive and bioresorbable compounds. There have been a number of important advances in this field during the past 30–40 years. Namely, by structural and compositional control, it became possible to choose whether calcium orthophosphate bioceramics were biologically stable once incorporated within the skeletal structure or whether they were resorbed over time. At the turn of the millennium, a new concept of calcium orthophosphate bioceramics—which is able to promote regeneration of bones—was developed. Presently, calcium orthophosphate bioceramics are available in the form of particulates, blocks, cements, coatings, customized designs for specific applications and as injectable composites in a polymer carrier. Current biomedical applications include artificial replacements for hips, knees, teeth, tendons and ligaments, as well as repair for periodontal disease, maxillofacial reconstruction, augmentation and stabilization of the jawbone, spinal fusion and bone fillers after tumor surgery. Exploratory studies demonstrate potential applications of calcium orthophosphate bioceramics as scaffolds, drug delivery systems, as well as carriers of growth factors, bioactive peptides and/or various types of cells for tissue engineering purposes.

## 1. Introduction

One of the most exciting and rewarding research areas of material science involves various applications to health care. Examples are sutures, catheters, heart valves, pacemakers, breast implants, fracture fixation plates, nails and screws in orthopedics, dental filling materials, orthodontic wires, as well as total joint replacement prostheses. Furthermore, during recent decades, both an ageing population and a democratization of high-risk sports have led to a surge of bone-related diseases and bone fractures, which must be treated. However, in order to be accepted by the living body, all implantable items must be prepared from a special class of materials, called biomedical materials or biomaterials, in short.

In general, all solids are divided into four major groups of materials: metals, polymers ceramics and composites thereof. Similarly, all biomaterials are also divided into the same major groups: biometals, biopolymers, bioceramics and biocomposites. All of them play very important roles in replacement and regeneration of human tissues. However, due to a great number of publications, this review is limited to bioceramics only. In general, the modern bioceramics comprise various polycrystalline materials, glasses, glass-ceramics, as well as ceramic-filled bioactive composites. All of them might be manufactured in both porous and dense forms in bulk, as well as in the forms of powders, granules and/or coatings. An expansion of bioceramics to health care has been characterized by a significant increase in the number of publications and patents in this field and an ever-increasing number of major international conferences and themed meetings [1,2,3,4,5].

**Figure 1:**
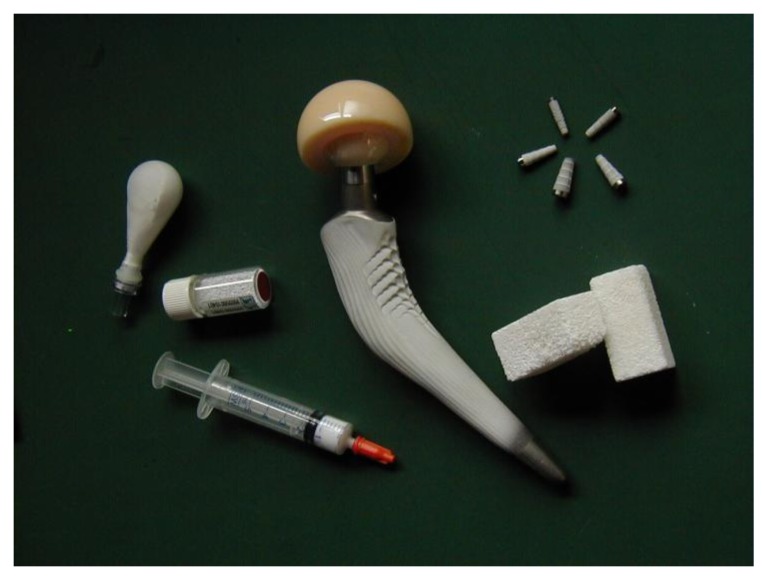
Several examples of the commercial calcium orthophosphate-based bioceramics.

Interestingly, the chemical elements used to manufacture bioceramics form just a small set of the Periodic Table. Namely, bioceramics might be prepared from alumina, zirconia, carbon, silica-contained and calcium-contained compounds, as well as some other chemicals [3]; however, this review is limited to calcium orthophosphates only. Calcium orthophosphate-based biomaterials and bioceramics are now used for a number of different applications throughout the body, covering all areas of the skeleton. Applications include dental implants, percutaneous devices and use in periodontal treatment, healing of bone defects, fracture treatment, total joint replacement (bone augmentation), orthopedics, cranio-maxillofacial reconstruction, otolaryngology and spinal surgery [2,3,4,5,6]. Depending upon the required properties, different calcium orthophosphates might be used. For example, Figure 1 shows some randomly chosen samples of the commercially available calcium orthophosphate bioceramics for bone graft applications.

In this review, the focus has been placed upon applications of calcium orthophosphates as medical implants to repair and reconstruct damaged or diseased hard tissues of the body (usually, those of the musculo-skeletal system, such as bones or teeth) and to describe some of the major developments in this field during the past ~40 years. To narrow the subject further, with a few important exceptions, bioceramics prepared from undoped and un-substituted calcium orthophosphates have been considered and discussed only. Furthermore, calcium orthophosphate bioceramics prepared from biological resources, such as bones, teeth, corals, *etc*., are not considered either. Readers interested in these topics are advised to read the original papers [7,8,9,10,11,12,13,14,15,16,17,18,19,20,21,22,23,24,25,26,27,28,29,30,31,32,33,34,35,36,37].

## 2. General Knowledge on Biomaterials and Bioceramics

A number of definitions have been developed for the term “biomaterials”. Until recently, the consensus definition developed by the experts in this field has been the following: biomaterials are synthetic or natural materials used to replace parts of a living system or to function in intimate contact with living tissues [38]. However, in September 2009, a more advanced definition was introduced: “A biomaterial is a substance that has been engineered to take a form which, alone or as part of a complex system, is used to direct, by control of interactions with components of living systems, the course of any therapeutic or diagnostic procedure, in human or veterinary medicine” [39]. In any case, biomaterials are intended to interface with biological systems to evaluate, treat, augment or replace any tissue, organ or function of the body and are now used in a number of different applications throughout the body [4,5,40]. The major difference between biomaterials and other classes of materials is the ability of biomaterials to remain in a biological environment without damaging the surroundings and without being damaged themselves in the process. Thus, biomaterials are solely associated with the health care domain and must have an interface with tissues or tissue components. One should stress that any artificial materials that are simply in contact with skin, such as hearing aids and wearable artificial limbs, are not included in the definition of biomaterials since the skin acts as a protective barrier between the body and the external world.

The biomaterials discipline is founded in the knowledge of the synergistic interaction of material science, biological science, chemical science, medical science and mechanical science and requires input and comprehension from all these areas so that implanted biomaterials perform adequately in a living body and interrupt normal body functions as little as possible [41]. As biomaterials mainly deal with all aspects of material synthesis and processing, the knowledge in chemistry, material science and engineering is essential. On the other hand, as clinical applications are the main purposes of biomaterials, biomedical sciences become a key part of the research. These include cell and molecular biology, anatomy and animal and human physiology. The final aim is to achieve the ideal biological interaction of implanted biomaterials with living tissues of a host. In order to achieve these goals, several stages have to be performed, namely: material synthesis, design and manufacturing of prostheses, followed by various types of tests. Furthermore, any potential biomaterial must also pass all regulatory requirements before its clinical application [42].

Biomaterials must be distinguished from *biological materials* because the former are the materials that are accepted by living tissues and, therefore, they might be used for tissue replacements, while the latter are the materials being produced by various biological systems (wood, cotton, bones, chitin, *etc.*) [43]. In addition, there are *biomimetic materials*, which are not made by living organisms but have similar composition, structure and properties to biological materials. Further, *bioceramics* (or biomedical ceramics) might be defined as biomaterials of the ceramic origin [44]. In general, bioceramics can have structural functions as joint or tissue replacements, can be used as coatings to improve the biocompatibility [45] of metal implants, as well as function as resorbable lattices, providing temporary structures and frameworks those are dissolved and/or replaced as the body rebuilds the damaged tissues [46,47,48,49,50,51]. Some types of bioceramics even feature a drug-delivery capability [52,53].

A progressive deterioration of all tissues with age is the major contributor to the need for spare parts for the body. Bone is especially vulnerable to fracture in older people due to a loss of density and strength with age. This effect is especially severe in women due to the hormonal changes associated with menopause. A graphical representation of the effect of time on bone strength and density from the age of 30 years onward is available in literature [Ref. 48, Figure 1]. Bone density decreases because bone-growing cells (osteoblasts) become progressively less productive in making new bone and repairing micro-fractures. The lower density greatly deteriorates the strength of bones and an unfortunate consequence is that many old people fracture their hips or have collapsed vertebrae and spinal problems [48].

Surface reactivity is one of the common characteristics of bioceramics. It contributes to their bone bonding ability and their enhancing effect on bone tissue formation. During implantation, various reactions occur at the material/tissue interfaces that lead to time-dependent changes in the surface characteristics of the implanted bioceramics and the surrounding tissues [54]. Bioceramics are needed to alleviate pain and restore functions to diseased or damaged calcified tissues (bones and teeth) of the body. A great challenge facing the medical application of bioceramics is to replace old, deteriorating bone with a material that can function the remaining years of the patient’s life and, ideally, be replaced by a new mature bone without transient loss of mechanical support [1]. Because the average life span of humans is now 80+ years and the major need for spare parts begins at about 60 years of age, the implanted non-resorbable bioceramics need to last, at least, for 20+ years. This demanding requirement of survivability is under conditions of use that are especially harsh to implanted materials: corrosive saline solutions at 37 °C under variable, multiaxial and cyclical mechanical loads. The excellent performance of the specially designed bioceramics that have survived these clinical conditions represents one of the most remarkable accomplishments of research, development, production and quality assurance during the past century [48].

**Table 1:** Existing calcium orthophosphates and their major properties [57,58].

Ca/P molar ratio	Compound	Formula	Solubility at 25 °C, −log(K_s_)	Solubility at 25 °C, g/L	pH stability range in aqueous solutions at 25 °C
0.5	Monocalcium phosphate monohydrate (MCPM)	Ca(H_2_PO_4_)_2_·H_2_O	1.14	~18	0.0–2.0
0.5	Monocalcium phosphate anhydrous (MCPA)	Ca(H_2_PO_4_)_2_	1.14	~17	^[c]^
1.0	Dicalcium phosphate dihydrate (DCPD), mineral brushite	CaHPO_4_·2H_2_O	6.59	~0.088	2.0–6.0
1.0	Dicalcium phosphate anhydrous (DCPA), mineral monetite	CaHPO_4_	6.90	~0.048	^[c]^
1.33	Octacalcium phosphate (OCP)	Ca_8_(HPO_4_)_2_(PO_4_)_4_·5H_2_O	96.6	~0.0081	5.5–7.0
1.5	α-Tricalcium phosphate (α-TCP)	α-Ca_3_(PO_4_)_2_	25.5	~0.0025	^[a]^
1.5	β-Tricalcium phosphate (β-TCP)	β-Ca_3_(PO_4_)_2_	28.9	~0.0005	^[a]^
1.0–2.2	Amorphous calcium phosphate (ACP)	Ca_x_H_y_(PO_4_)_z_·nH_2_O, n = 3–4.5; 15–20% H_2_O	^[b]^	^[b]^	~5–12^ [d]^
1.5–1.67	Calcium-deficient hydroxyapatite (CDHA)^[e]^	Ca_10-*x*_(HPO_4_)*_x_*(PO_4_)_6-*x*_(OH)_2-*x*_^[f]^ (0 < *x* < 1)	~85.1	~0.0094	6.5–9.5
1.67	Hydroxyapatite (HA, HAp or OHAp)	Ca_10_(PO_4_)_6_(OH)_2_	116.8	~0.0003	9.5–12
1.67	Fluorapatite (FA or FAp)	Ca_10_(PO_4_)_6_F_2_	120.0	~0.0002	7–12
1.67	Oxyapatite (OA or OAp)	Ca_10_(PO_4_)_6_O	~69	~0.087	^[a]^
2.0	Tetracalcium phosphate (TTCP or TetCP), mineral hilgenstockite	Ca_4_(PO_4_)_2_O	38–44	~0.0007	^[a]^

^[a]^ These compounds cannot be precipitated from aqueous solutions. ^[b]^ Cannot be measured precisely. However, the following values were found: 25.7 ± 0.1 (pH = 7.40), 29.9 ± 0.1 (pH = 6.00), 32.7 ± 0.1 (pH = 5.28). The comparative extent of dissolution in acidic buffer is: ACP >> α-TCP >> β-TCP > CDHA >> HA > FA. ^[c]^ Stable at temperatures above 100 °C. ^[d]^ Always metastable. ^[e]^ Occasionally CDHA is named as precipitated HA. ^[f]^ In the case *x* = 1 (the boundary condition with Ca/P = 1.5), the chemical formula of CDHA looks as follows: Ca_9_(HPO_4_)(PO_4_)_5_(OH).

## 3. General Knowledge on Calcium Orthophosphates

The main driving force behind the use of calcium orthophosphates as bone substitute materials is their chemical similarity to the mineral component of mammalian bones and teeth [55,56,57,58]. As a result, in addition to being non-toxic, they are biocompatible, not recognized as foreign materials in the body and, most importantly, exhibit both bioactive behavior [59] and integrate into living tissue by the same processes active in remodeling healthy bone. This leads to an intimate physicochemical bond between the implants and bones, termed osteointegration [60]. More to the point, calcium orthophosphates are also known to be osteoconductive (able to provide a scaffold or template for new bone formation) and support osteoblast adhesion and proliferation [61,62]. Even so, the major limitations to use calcium orthophosphates as load-bearing bioceramics are their mechanical properties; namely, they are brittle with a poor fatigue resistance [46,47,48,63]. The poor mechanical behavior is even more evident for highly porous bioceramics and scaffolds because porosity greater than ~100 µm is considered as the requirement for proper vascularization and bone cell colonization [64,65,66]. Thus, for biomedical applications, calcium orthophosphates are used primarily as fillers and coatings, rendering it impossible to use them for repair of large osseous defects [57,58].

The complete list of known calcium orthophosphates, including their standard abbreviations and the major properties, is given in Table 1, while detailed information on their synthesis, structure, chemistry, other properties and biomedical application has been comprehensively reviewed recently [57,58]; interested readers are referred here. Additional thorough information on various calcium orthophosphates can be found in books and monographs [67,68,69,70,71,72,73,74,75]. One should note that among the existing calcium orthophosphates (Table 1), only certain compounds are useful for biomedical applications, because those having a Ca/P ionic ratio less than 1 are not suitable for implantation due to their high solubility and acidity. Due to its basicity, TTCP is not suitable either. However, to be used in medicine, the “unsuitable” calcium orthophosphates might successfully be combined with either other calcium orthophosphates or other chemicals.

## 4. Bioceramics of Calcium Orthophosphates

### 4.1. History

The performance of living tissues is the result of millions of years of evolution, while the performance of acceptable artificial substitutions that humankind has designed to repair damaged tissues are only a few decades old. However, attempts to repair the human body with the use of implant materials are recorded in the early medical writings of the Hindu, Egyptian and Greek civilizations. The earliest successful implants were in the skeletal system. Historically, selection of the materials was based on their availability and an ingenuity of the individual making and applying the prosthetic [76]. Archaeological findings exhibited in museums showed that materials used to replace missing human bones and teeth included animal or human (from corpses) bones and teeth, shells, corals, ivory (elephant tusk), wood, as well as some metals (gold or silver). For instance, the Etruscans learned to substitute missing teeth with bridges made from artificial teeth carved from the bones of oxen, while in ancient Phoenicia loose teeth were bound together with gold wires, tying artificial ones to neighboring teeth. In the 17th century, a piece of dog skull was successfully transplanted into the damaged skull of a Dutch duke. The Chinese recorded the first use of dental amalgam to repair decayed teeth in the year 659 AD, while pre-Columbian civilizations used gold sheets to heal cranial cavities following trepanation [77]. Furthermore, in 1970, Amadeo Bobbio discovered Mayan skulls, some of then more than ~4000 years old, in which missing teeth had been replaced by nacre substitutes [78]. Unfortunately, due to the practice of cremation in many societies, little is known about prehistoric materials used to replace bone lost to accident or disease.

The first widely tested artificial bioceramic was plaster of Paris. However, in the past, many implantations failed due to infections, which tended to be exacerbated in the presence of implants, since they provided a region inaccessible to the body’s immunologically competent cells. Thus, the use of biomaterials did not become practical until the advent of an aseptic surgical technique developed by J. Lister in the 1860s. Furthermore, there was a lack of knowledge about the toxicity of selected materials. In this frame, application of calcium orthophosphates appears to be logical due to their similarity with the mineral phases of bones and teeth [55,56,69,79,80]. Calcium orthophosphates are not toxic and do not cause cell death in the surrounding tissues. However, according to available literature, the first attempt to use them (it was TCP) as an artificial material to repair surgically created defects in rabbits was performed in 1920 [81]. Although this may be the first scientific study on use of a calcium orthophosphate for bone defects repair, it remains unclear whether the calcium orthophosphate was a precipitated or a ceramic material and whether it was in a powder or granular form. The second clinical report was published 30 years later [82]. More than 20 years afterwards, the first dental application of a calcium orthophosphate (erroneously described as TCP) in surgically created periodontal defects [83] and the use of dense HA cylinders for immediate tooth root replacement [84] were reported. According to the available databases, the first paper with the term “bioceramics” in the abstract was published in 1971 [85], and with the term in the title in 1972 [86,87]. However, application of the ceramic materials as prostheses had been known before [88,89,90,91]. Further historical details might be found in literature [92,93]. On April 26, 1988, the first international symposium on bioceramics was held in Kyoto, Japan.

Commercialization of the dental and surgical applications of calcium orthophosphate (mainly, HA) bioceramics occurred in the 1980s, largely due to the pioneering efforts by Jarcho [94,95,96,97] in the USA, De Groot [67,98,99] in Europe and Aoki [100,101,102,103] in Japan. Shortly afterwards, HA became a bioceramic of reference in the field of calcium orthophosphates for biomedical applications. Preparation and biomedical applications of apatites derived from sea corals (coralline HA) [104,105,106] and bovine bone [107] were reported at the same time [108].

### 4.2. Chemical Composition and Preparation

Currently, calcium orthophosphate bioceramics can be prepared from various sources [109,110,111,112,113,114,115,116]. Unfortunately, up until now, all attempts to synthesize bone replacement materials for clinical applications featuring physiological tolerance, biocompatibility and a long-term stability have had only relative success; showing the superiority and a complexity of the natural structures [117].

In general, calcium orthophosphate bioceramics should be characterized from many viewpoints such as the chemical composition (stoichiometry and purity), homogeneity, phase distribution, morphology, grain sizes and shape, grain boundaries, crystallite size, crystallinity, pores, cracks, surface, *etc*. From the chemical point of view, the vast majority of calcium orthophosphate bioceramics is based on HA, β-TCP, α-TCP and/or biphasic calcium phosphate (BCP, which is an intimate mixture of either β-TCP + HA [118,119,120,121,122,123,124,125,126,127,128,129,130] or α-TCP + HA [7,8,9,10,11]) [131,132,133,134,135,136,137,138,139]. One should note that recently the concept of BCP has been extended by preparation and characterization of biphasic TCP, consisting of α-TCP and β-TCP phases [140,141,142,143,144]. The biphasic TCP is usually prepared by heating ACP precursors [142,143,144], in which the α-TCP/β-TCP ratio can be controlled by aging time and pH value during synthesis of the amorphous precursor [143]. Furthermore, very recently, a triphasic formulation, consisting of HA, α-TCP and β-TCP has been prepared [145]. The preparation techniques of various calcium orthophosphates have been extensively reviewed in literature [57,58,67,68,69,70,71,72,73,74,75] and references therein. When compared to both α- and β-TCP, HA is a more stable phase under the physiological conditions, as it has a lower solubility (Table 1) and, thus, a slower resorption kinetics [69,131,132]. Therefore, the BCP concept is determined by the optimum balance of a more stable phase of HA and a more soluble TCP. Due to a higher biodegradability of the α- or β-TCP component, the reactivity of BCP increases with increasing TCP/HA ratio. Thus, *in vivo* bioresorbability of BCP can be controlled through the phase composition [127]. Similar conclusions are also valid for both the biphasic TCP (in which α-TCP is a more soluble phase) and the triphasic (HA, α-TCP and β-TCP) formulation.

As implants made of calcined HA are found in bone defects for many years after implantation, bioceramics made of more soluble calcium orthophosphates [7,8,9,10,11,118,119,120,121,122,123,124,125,126,127,128,129,130,133,134,135,136,137,138,139,140,141,142,143,144,145,146,147] are preferable for biomedical purposes. Furthermore, experimental results showed that BCP had a higher ability to adsorb fibrinogen, insulin or type I collagen than HA [148]. Thus, according to both observed and measured bone formation parameters, calcium orthophosphates have been ranked as follows: low sintering temperature BCP (rough and smooth) ≈medium sintering temperature BCP ≈ TCP > calcined low sintering temperature HA > non-calcined low sintering temperature HA > high sintering temperature BCP (rough and smooth) > high sintering temperature HA (calcined and non-calcined) [149]. This sequence was developed in 2000 and, thus, neither biphasic TCP, nor triphasic (HA, α-TCP and β-TCP) formulation have been included. Recent developments in processing and surface modification of HA have been reviewed elsewhere [150].

### 4.3. Forming and Shaping

In order to fabricate bioceramics in more and more complex shapes, scientists are investigating the use of old and new manufacturing techniques. These techniques range from an adaptation of age-old pottery techniques to the latest manufacturing methods for high-temperature ceramic parts for airplane engines. For example, reverse engineering and rapid prototyping technologies have revolutionized a generation of physical models, allowing an engineer to efficiently and accurately produce physical models and customized implants with high levels of geometric intricacy [151,152,153]. Combined with the computer-aided design and manufacturing (CAD/CAM), complex physical objects of the anatomical structure can be fabricated in a variety of sizes. In a typical application, an image of a bone defect in a patient can be taken and used to develop a three-dimensional (3D) CAD computer model [154,155,156]. A computer can then reduce the model to slices or layers. The 3D objects are constructed layer-by-layer using rapid prototyping techniques such as fused deposition modeling [157,158], selective laser sintering [159,160,161], 3D printing [162,163,164,165,166,167,168,169,170] or stereo lithography [171,172,173,174]. A custom-made implant of actual dimensions would reduce the time it takes to perform the medical implantation procedure and subsequently lower the risk to the patient. Another advantage of a prefabricated, exact-fitting implant is that it can be used more effectively and applied directly to the damaged site rather than a replacement that is formulated during surgery from a paste or granular material [175,176,177]. In some cases, laser processing can be applied as well [178].

The manufacturing technique depends greatly on the ultimate application of the bioceramic device, whether it is for a hard-tissue replacement or integration of the device within the surrounding tissues. In general, three types of processing technologies are used: (1) employment of a lubricant and a liquid binder with ceramic powders for shaping and subsequent firing; (2) application of self-setting and self-hardening properties of water-wet molded powders (cementation); (3) melting of materials to form a liquid and shaping during cooling and solidification [179,180,181,182]. Since calcium orthophosphates are either thermally unstable (MCPM, MCPA, DCPA, DCPD, OCP, ACP, CDHA) or have a melting point at temperatures exceeding ~1400 °C (α-TCP, β-TCP, HA, FA, TTCP), only the first and second consolidation approaches are used to prepare bulk bioceramics and scaffolds. The methods include uniaxial compaction [183,184], isostatic pressing (cold or hot) [185,186,187,188,189,190,191], granulation [192], loose packing [193], slip casting [194,195,196], gel casting [173,174,197,198,199,200,201,202], pressure mold forming [203], injection molding [204], polymer replication [205,206,207,208], extrusion [209,210,211], slurry dipping and spraying [212]. In addition, formation of ceramic sheets from slurries tape casting [130,199,213,214], doctor blade [215] and colander methods might be employed [63,179,180,181,182]. Furthermore, some of these processes might be performed under the magnetic field, which helps crystal aligning [216,217,218,219].

Powders are usually pressed damp in metal dies or dry in lubricated dies at pressures high enough to form sufficiently strong structures to hold together until they are sintered. An organic binder such as polyvinyl alcohol helps to bind the powder together [185]. Drying at about 100 °C is a critical step in preparing damp-formed pieces for firing. Too much or too little water in the compacts can lead to blowing apart the ware on heating or crumbling, respectively. The binder is removed by heating in air to oxidize the organic phases to carbon dioxide and water [179,180,181,182].

Furthermore, forming and shaping of any ceramic products require a proper selection of the raw materials in terms of particle sizes and size distribution. Namely, tough and strong bioceramics consist of pure, fine and homogeneous microstructures. To attain this, pure powders with small average size and high surface area must be used as the starting sources. However, for maximum packing and least shrinkage after firing, mixing of ~70% coarse and ~30% fine powders have been suggested [182]. Mixing is usually carried out in a ball mill for uniformity of properties and reaction during subsequent firing. Mechanical die forming, or sometimes extrusion through a die orifice, can be used to produce a fixed cross-section. Drying involves removal of water and subsequent shrinkage of the product. However, due to local variations in water content, warping and even cracks may be developed during drying. Dry pressing and hydrostatic molding can minimize these problems [182]. Afterwards, the manufactured green samples are sintered.

Finally, to produce the accurate shaping, necessary for the fine design of bioceramics, machine finishing might be essential [156,179,220]. Unfortunately, cutting tools developed for metals are usually useless for bioceramics due to their fragility; therefore, grinding and polishing appear to be the convenient finishing techniques [156,179]. Furthermore, the surface of bioceramics might be modified by various additional treatments [221].

### 4.4. Sintering and Firing

**Figure 2:**
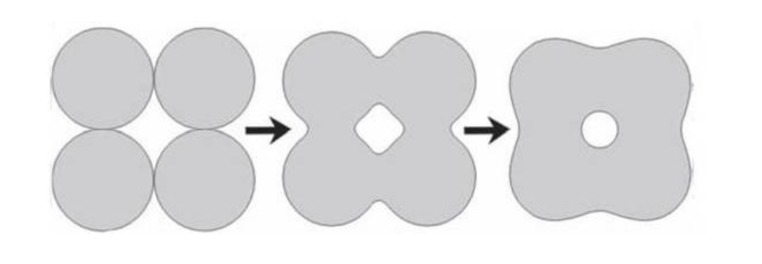
A schematic diagram representing the changes occurring with particles under sintering.

A sintering (or firing) procedure appears to be of a great importance to manufacture bulk bioceramics with the required properties. Usually, this stage is carried out according to controlled temperature programs of electric furnaces in adjusted ambience of air with necessary additional gasses; however, always at temperatures below the melting points of the materials. The firing step can include temporary holds at intermediate temperatures to burn out organic binders [179,180,181,182]. The heating rate, sintering temperature and holding time depend on the starting materials. For example, in the case of HA, these values are in the ranges of 0.5–3 °C/min, 1000–1250 °C and 2–5 h, respectively [222]. In the majority cases, sintering allows a structure to retain its shape. However, this process might be accompanied by a considerable degree of shrinkage [107], which must be accommodated in the fabrication process. The sintering mechanism is controlled by both surface and volume diffusion at grain boundaries. In general, when solids heat to high temperatures, the constituent ions or atoms are driven to move to fill up pores and open channels between the grains of powders, as well as to compensate for the surface energy differences among their convex and concave surfaces. At the initial stages, bottlenecks are formed and grow among the particles (Figure 2). Existing vacancies tend to flow away from the surfaces of sharply curved necks; this is an equivalent of a material flow towards the necks, which grow as the voids shrink. Small contact areas among the particles expand and, at the same time, a density of the compact increases and the total void volume decreases. As the pores and open channels are closed during a heat treatment, the particles become tightly bonded together and density, strength and fatigue resistance of the sintered object improve greatly. Grain-boundary diffusion was identified as the dominant mechanism for densification [223]. Furthermore, strong chemical bonds form among the particles and loosely compacted green bodies are hardened to denser materials [179,180,181,182].

**Figure 3:**
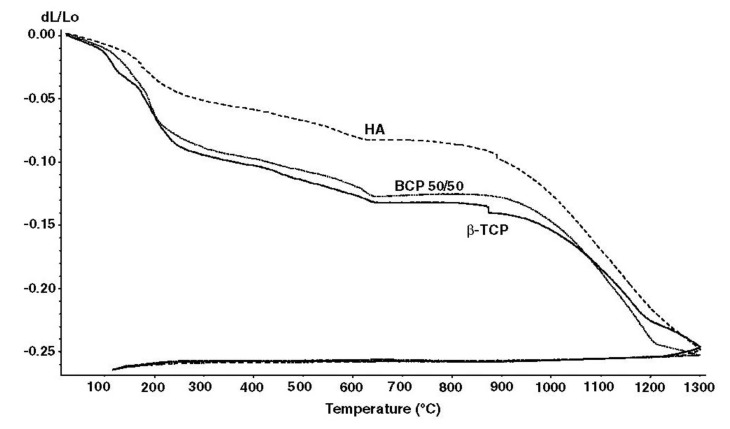
Linear shrinkage of the compacted ACP powders that were converted into β-TCP, BCP (50% HA + 50% β-TCP) and HA upon heating. According to the authors: “At 1300 °C, the shrinkage reached a maximum of approximately ~25, ~30 and ~35% for the compacted ACP powders that converted into HA, BCP 50/50 and β-TCP, respectively” [224]. Reprinted from [224] with permission.

In the case of calcium orthophosphates, several specific processes occur during sintering. Firstly, moisture, carbonates and all other volatile chemicals remaining from the synthesis stage, such as ammonia, nitrates and any organic compounds, are removed as gaseous products. Secondly, unless powders are sintered, the removal of these gases facilitates production of denser ceramics with subsequent shrinkage of the samples (Figure 3). Thirdly, all chemical changes are accompanied by a concurrent increase in crystal size and a decrease in the specific surface area. Fourthly, a chemical decomposition of all acidic orthophosphates and their transformation into other phosphates (e.g., 2HPO_4_^2−^ → P_2_O_7_^4−^ + H_2_O↑) takes place.

In addition, sintering causes toughening [225], densification [226], as well as increasing the mechanical strength [227,228]. The latter events are due to presence of air and other gases filling gaps among the unsintered powders. At sintering, the gases move towards the outside of powders and green bodies shrink owing to decrease of distances among powders. However, in the case of FA sintering, a linear shrinkage was found to occur at ~715 °C and the material reached its final density at ~890 °C. Above this value, grain growth became important and induced an intra-granular porosity, which was responsible for density decrease. At ~1180 °C, a liquid phase forms due to formation of a binary eutectic between FA and fluorite contained in the powder as impurity. This liquid phase further promoted the coarsening process and induced formation of large pores at high temperatures [229]. Sintering of a biologically formed apatite has been investigated [230,231] as well, and the obtained products have been characterized [232,233]. In all cases, the numerical value of the Ca/P ratio in sintered apatites of biological origin was higher than that of the stoichiometric HA. One should mention that in the vast majority of cases, calcium orthophosphates with Ca/P ratio < 1.5 (Table 1) are not sintered, since these compounds are thermally unstable, while sintering of CDHA and ACP leads to their transformation into BCP (HA + β-TCP [234] or HA + α-TCP [235]).

An extensive study on the effects of sintering temperature and time on the properties of HA bioceramics revealed a correlation between these parameters and density, porosity, grain size, chemical composition and strength of the scaffolds [236]. Namely, sintering below ~1000 °C was found to result in initial particle coalescence, with little or no densification and a significant loss of the surface area and porosity. The degree of densification appeared to depend on the sintering temperature, whereas the degree of ionic diffusion was governed by the period of sintering [236]. Furthermore, various sintering additives might be added to calcium orthophosphate bioceramics to enhance sinterability [237,238,239,240]. Unexpectedly, a magnetic field during sintering was found to influence the growth of HA grains [241].

HA powders can be pressurelessly sintered up to the theoretical density at 1000–1200 °C. Processing at higher temperatures may lead to exaggerated grain growth and decomposition because HA becomes unstable at temperatures exceeding ~1300 °C [67,68,69,70,71,72,73,74,75,242]. The decomposition temperature of HA bioceramics is a function of the partial pressure of water vapor. Moreover, processing under vacuum leads to an earlier decomposition of HA, while processing under high partial pressure of water prevents the decomposition. On the other hand, the presence of water in the sintering atmosphere was reported to inhibit densification of HA and accelerate grain growth [63,243]. A definite correlation between hardness, density and grain size in sintered HA bioceramics was found: despite exhibiting high bulk density, hardness started to decrease at a certain critical grain size limit [244,245].

Hot pressing [245,246,247,248,249,250,251], hot isostatic pressing (HIP) [189,190] or hot pressing with post-sintering [252,253] processes make it possible to decrease the temperature of the densification process, diminish the grain size, as well as achieve higher densities. This leads to finer microstructures, higher thermal stability of calcium orthophosphates and subsequently better mechanical properties of bulk bioceramics. Microwave [254,255,256,257,258,259,260,261] and spark plasma [262,263,264,265,266,267,268,269,270] sintering techniques are alternative methods to the conventional sintering, hot pressing and HIP. Both techniques were found to be time and energy efficient densification methods. Recently, a hydrothermal hot pressing method was developed to fabricate OCP bioceramics without thermal dehydration and/or thermal decomposition [271]. Further details on the sintering and firing processes of calcium orthophosphate bioceramics are available in literature [47,63,69,70,272,273].

To conclude this part, one should mention an excellent recent review on various ceramic manufacturing techniques [274], to which interested readers are referred to extend their knowledge on ceramic processing.

## 5. The Major Properties

### 5.1. Mechanical Properties

Ideally, a bone substitute should be replaced by a mature bone without transient loss of mechanical support. Unfortunately for material scientists, a human body provides one of the most inhospitable environments for implanted materials. It is warm, wet and both chemically and biologically active. Furthermore, the body is capable of generating quite massive force concentrations and the variance in such characteristics among individuals might be enormous. Therefore, all types of potential biomaterials and bioceramics must sustain attacks of a great variety of aggressive conditions. Regrettably, there is presently no material fulfilling all these requirements.

On the other hand, any ceramics, when they fail, tend to do so in a dramatic manner. Namely, the brittle nature of calcium orthophosphate bioceramics is attributed to high strength ionic bonds. Thus, it is not possible for plastic deformation to happen prior to failure, as a slip cannot occur. Consequently, if a crack is initiated, its progress will not be hindered by the deformation of material ahead of the crack, as would be the case in a ductile material (e.g., a metal). The crack will continue to propagate, rapidly resulting in a catastrophic failure [180].

Accordingly, from the mechanical point of view, calcium orthophosphate bioceramics appear to be brittle polycrystalline materials for which the mechanical properties are governed by crystallinity, grain size, grain boundaries, porosity and composition [188]. It appears to be very sensitive to slow crack growth [275]. For dense bioceramics, the strength is a function of the grain size. Finer grain size materials have smaller flaws at the grain boundaries and thus are stronger than bioceramics with larger grain sizes. In general, the mechanical properties decrease significantly with increasing content of an amorphous phase, microporosity and grain size, while a high crystallinity, a low porosity and small grain size tend to give a higher stiffness, a higher compressive and tensile strength and a greater fracture toughness. Thus, calcium orthophosphate bioceramics possess poor mechanical properties (for instance, a low impact and fracture resistances) that do not allow use in load-bearing areas, such as artificial teeth or bones [46,47,48,49,50,51,52,276]. For example, fracture toughness [277] of HA bioceramics does not exceed ~1.2 MPa·m^1/2^ [278] (human bone: 2–12 MPa·m^1/2^). It decreases almost linearly with increasing porosity [63]. Generally, fracture toughness increases with decreasing grain size. However, in some materials, especially non-cubic ceramics, fracture toughness reaches the maximum and rapidly drops with decreasing grain size. For example, Halouani *et al.* investigated fracture toughness of pure hot pressed HA with grain sizes of 0.2–1.2 µm [251]. There appeared to be two distinct trends, where fracture toughness decreased with increasing grain size above ~0.4 µm and subsequently decreased with decreasing grain size. The maximum fracture toughness measured was 1.20 ± 0.05 MPa·m^1/2^ at ~0.4 µm [251]. Fracture energy of HA bioceramics is in the range of 2.3–20 J/m^2^, while the Weibull modulus [279] is low (~5–12) in wet environments, which means that HA behaves as a typical brittle ceramics and indicates low reliability of HA implants [63]. Interestingly, three peaks of internal friction were found at temperatures about −40, 80 and 130 °C for HA but no internal friction peaks were obtained for FA in the measured temperature range; this effect was attributed to the differences of the positions of F^-^ and OH^-^ in FA and HA, respectively [280].

Bending, compressive and tensile strengths of dense HA bioceramics are in the range of 38–250 MPa, 120–900 MPa and 38–300 MPa, respectively. Similar values for porous HA bioceramics are in the range of 2–11 MPa, 2–100 MPa and ~3 MPa, respectively [63]. These wide variations in the properties are due to both structural variations (e.g., an influence of remaining microporosity, grain sizes, presence of impurities, *etc*.) and manufacturing processes, as well as caused by a statistical nature of the strength distribution. Strength [281] was found to increase with increasing Ca/P ratio, reaching a maximum value around Ca/P ~1.67 (stoichiometric HA) and to decrease suddenly when Ca/P > 1.67 [63]. Furthermore, strength decreases almost exponentially with increasing porosity [119,120]. However, by changing the pore geometry, it is possible to influence the strength of porous bioceramics. It is also worth mentioning that porous HA bioceramics are considerably less fatigue [282] resistant than dense ones. Both grain sizes and porosity are reported to influence the fracture path, which itself has little effect on the fracture toughness of calcium orthophosphate bioceramics [188,283]. Furthermore, no obvious decrease in mechanical properties was found after calcium orthophosphate bioceramics had been aged in various solutions for different time periods [284].

Young’s (or elastic) modulus [285] of dense HA bioceramics is in the range of 35–120 GPa, which is more or less similar to those of the most resistant components of the natural calcified tissues (dental enamel: ~74 GPa, dentine: ~21 GPa, compact bone: ~18–22 GPa). Nevertheless, dense bulk compacts of HA have mechanical resistances of the order of 100 MPa *versus* ~300 MPa of human bones, diminishing drastically their resistance in the case of porous bulk compacts [286]. Young’s modulus measured in bending is between 44 and 88 GPa. Recently, a considerable anisotropy in the stress-strain behavior of the perfect HA crystals was found by *ab initio* calculations [287]. The crystals appeared to be brittle for tension along the *z*-axis with the maximum stress of ~9.6 GPa at 10% strain. Furthermore, the structural analysis of the HA crystal under various stages of tensile strain revealed that the deformation behavior manifested itself mainly in the rotation of PO_4_ tetrahedrons with concomitant movements of both the columnar and axial Ca ions [287]. Vickers hardness [288] of dense HA bioceramics is within 3–7 GPa, while the Poisson’s ratio [289] for the synthetic HA is about 0.27, which is close to that of bones (~0.3). At temperatures within 1000–1100 °C, dense HA bioceramics were found to exhibit superplasticity with a deformation mechanism based on grain boundary sliding. Furthermore, both the wear resistance and friction coefficient of dense HA bioceramics are comparable to those of dental enamel [63].

Due to high brittleness (associated to a low crack resistance), biomedical applications of calcium orthophosphate bioceramics are focused on production of non-load-bearing implants, such as pieces for middle ear surgery, filling of bone defects in oral or orthopedic surgery, as well as coating of dental implants and metallic prosthesis (see below) [117,290,291]. In order to improve the reliability of calcium orthophosphate bioceramics, diverse reinforcements (ceramics, metals or polymers) have been applied to manufacture various biocomposites and hybrid biomaterials [292], but that is another story. However, successful hybrid formulations consisting of calcium orthophosphates only should be mentioned [293,294,295,296,297,298]. For example, bulk HA bioceramics might be reinforced by HA whiskers [294,295,296,297]. Furthermore, a superior superplasticity of HA/β-TCP composites (*i.e*., BCP) to HA bioceramics has been detected [298].

Another approach to improve the mechanical properties of calcium orthophosphate bioceramics is to coat the items by a polymeric layer [299,300]; however, this is still other story. Interested readers are referred to further details on the mechanical properties of calcium orthophosphate bioceramics available elsewhere [63,301].

### 5.2. Electrical Properties

Occasionally, interest is expressed in the electrical properties of calcium orthophosphate bioceramics. For example, a surface ionic conductivity of both porous and dense HA bioceramics was examined for humidity sensor applications, since the room temperature conductivity was influenced by relative humidity [302]. Namely, the ionic conductivity of HA has been a subject of research for its possible use as an alcohol [303], carbon dioxide [303] or carbon monoxide [304] gas sensors. Electrical measurements have also been used as a characterization tool to study the evolution of microstructure in HA bioceramics [305]. More to the point, Valdes *et al.* examined the dielectric properties of HA to understand its decomposition to β-TCP [306]. In the case of CDHA, the electrical properties, in terms of ionic conductivity, were found to increase after compression of the samples at 15 t/cm^2^, which was attributed to establishment of some order within the apatitic network [307]. The conductivity mechanism of CDHA appeared to be multiple [308]. Furthermore, there is an attempt to develop CDHA whisker electrets for biomedical utilization [309].

Interestingly, the electrical properties of calcium orthophosphate bioceramics appear to influence their biomedical applications. For example, there is an interest in polarization of HA bioceramics to generate a surface charge by the application of electric fields at elevated temperatures [310,311]. The presence of surface charges on HA bioceramics was shown to have a significant effect on both *in vitro* and *in vivo* crystallization of biological apatite [312,313,314,315,316]. Furthermore, growth of both biomimetic calcium orthophosphates and bones was found to be accelerated on negatively charged surfaces and decelerated on positively charged surfaces [316,317,318,319,320,321,322,323,324,325]. In addition, the electrical polarization of HA bioceramics was found to accelerate a cytoskeleton reorganization of osteoblast-like cells [326,327,328], extend bioactivity [329] and enhance bone ingrowth through the pores of porous HA implants [330]. There is an interesting study on the interaction of a blood coagulation factor on electrically polarized HA surfaces [331]. Further details on the electrical properties of calcium orthophosphate-based bioceramics can be found in [258,332,333,334,335,336].

### 5.3. Possible Transparency

Single crystals of all calcium orthophosphates are optically transparent for visible light. As bioceramics of calcium orthophosphates have a polycrystalline nature with a random orientation of big amounts of small crystals they are opaque and of white color, unless colored dopants have been added. However, in some cases, transparency is convenient to provide some essential advantages (e.g., to enable direct viewing of living cells in a transmitted light). Thus, transparent calcium orthophosphate bioceramics have been prepared and investigated [189,191,267,270,337,338,339,340,341,342,343,344]. The preparation techniques, for example, include hot isostatic pressing [189,191], ambient-pressure sintering [337], gel casting coupled with a low-temperature sintering [340,343], pulse electric current sintering [341], as well as spark plasma sintering [267,270]. Fully dense, transparent calcium orthophosphate bioceramics were obtained at temperatures above ~800 °C. Depending on the preparation technique, the transparent calcium orthophosphate bioceramics have a uniform grain size ranging from ~0.2 μm [337] to ~250 μm [340] and are always pore-free; the latter is not good for biomedical applications.

### 5.4. Porosity

**Figure 4:**
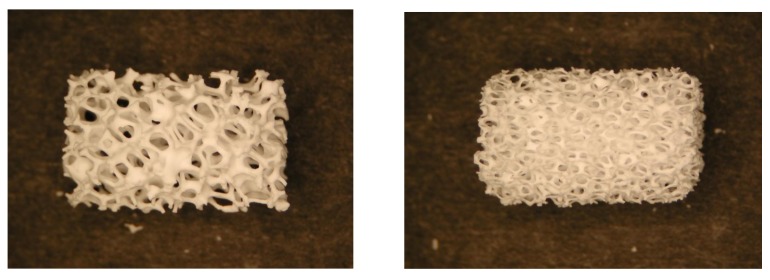
Photographs of a commercially available porous calcium orthophosphate bioceramic with different porosity. Horizontal field width is 20 mm.

**Figure 5:**
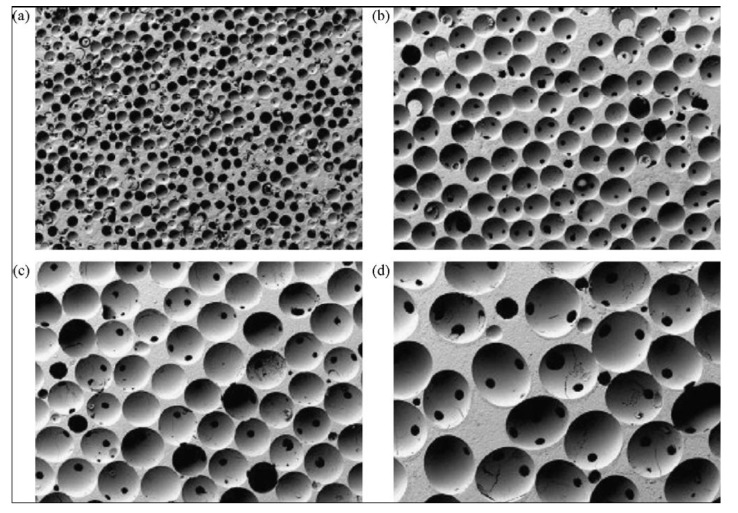
β-TCP porous ceramics with different pore sizes prepared using polymethylmethacrylate balls with the diameters: (**a**) 100–200; (**b**) 300–400; (**c**) 500–600 and (**d**) 700–800 μm. Horizontal field width is 45 mm. Reprinted from [377] with permission.

Porosity is defined as the percentage of void spaces in solids and it is a morphological property independent of the material. The surface area of porous bodies is much higher, which guarantees a good mechanical fixation in addition to providing sites on the surface that allow chemical bonding between the bioceramics and bones [345]. Furthermore, a porous material may have both closed (isolated) pores and open (connected) pores. Connected pores look like tunnels and are accessible by gases, liquids and particulate suspensions [346]. The open-cell nature of reticulated materials is a unique characteristic essential in many applications. Furthermore, dimensions of open pores are directly related to bone formation, since such pores grant both the surface and space for cell adhesion and bone ingrowth. On the other hand, pore interconnection provides the way for cell distribution and migration, as well as allowing efficient *in vivo* blood vessel formation suitable for sustaining bone tissue neo-formation and possibly remodeling [64,65,66,122,347,348,349,350,351,352,66,122,347]. Namely, porous HA bioceramics can be colonized by bone tissues [349,353,354,355,356,357,358,359,360,361,362,363]. Therefore, interconnecting macroporosity (pore size >100 μm) [118,345,349,364,365], which is defined by its capacity to be colonized by cells, is intentionally introduced in solid bioceramics (Figure 4). Macroporosity is usually formed due to a release of various volatile materials and, for that reason, incorporation of pore-creating additives (porogens) is the most popular technique to create macroporosity. The porogens are crystals or particles of either volatile (they evolve gases at elevated temperatures) or soluble substances, such as paraffin, naphthalene, sucrose, NaHCO_3_, gelatin, polymethylmethacrylate or even hydrogen peroxide [119,272,366,367,368,369,370,371,372,373]. Obviously, the ideal porogen should be nontoxic and be removed at ambient temperature, thereby allowing the ceramic/porogen mixture to be injected directly into a defect site and allowing the scaffold to fit the defect [374]. Sintering particles, preferably spheres of equal size, is a similar way to generate porous 3D bioceramics of calcium orthophosphates (Figure 5). However, pores resulting from this method are often irregular in size and shape and not fully interconnected with one another.

Several other techniques, such as replication of polymer foams by impregnation, dual-phase mixing, particulate leaching, freeze casting, slip casting, stereo lithography and foaming of gel casting suspensions, have been applied to fabricate porous calcium orthophosphate bioceramics [64,65,66,104,180,196,199,202,205,206,207,246,247,364,365,366,367,368,369,370,371,372,373,374,375,376,377,378,379,380,381,382,383,384,385,386,387,388,389,390,391,392,393,394,395,396,397,398,399,400,401,402,403,404,405,406,407,408]. Some are summarized in Table 2 [374]. Furthermore, natural porous materials, like coral skeletons made of CaCO_3_, can be converted into porous HA under hydrothermal conditions (250 °C, 24–48 h) with the microstructure undamaged [104,105,106]. Porous HA bioceramics can also be obtained by hydrothermal hot pressing. This technique allows solidification of the HA powder at 100–300 °C (30 MPa, 2 h) [381]. In another approach, bi-continuous water-filled microemulsions have been used as pre-organized systems for the fabrication of needle-like frameworks of crystalline HA (2 °C, three weeks) [382,383]. Porous HA bioceramics might be prepared by a combination of gel casting and foam burn out methods [202]. Lithography was used to print a polymeric material, followed by packing with HA and sintering [384]. A hot pressing technique can be applied as well [246,247]. In addition, an HA suspension can be cast into a porous CaCO_3_ skeleton, which is then dissolved, leaving a porous network [376]. 3D periodic macroporous frame of HA has been fabricated via a template-assisted colloidal processing technique [385]. Furthermore, porous HA bioceramics might be prepared by using different starting HA powders and sintering at various temperatures by pressureless-sintering method [391].

**Table 2:** The procedures used to manufacture porous calcium orthophosphate scaffolds for tissue engineering [374].

Year	Who and where	Process	Calcium orthophosphate	Sintering	Compressive strength	Pore sizes	Porosity
2006	Deville *et al.* Berkeley, CA	HA + ammonium methacrylate in PTFE mold, freeze dried and sintered.	HA	Yes: 1300 °C	16 MPa, 65 MPa, 145 MPa	open unidirectional 50–150 μm	>60%, 56%, 47%
2006	Saiz *et al.* Berkeley, CA	Polymer foams coated, compressed after infiltration, then calcined.	HA powder	Yes: 700–1300 °C	–	100–200 μm	–
2006	Murugan *et al.* Singapore + USA	Bovine bone cleaned, calcined.	Bovine bone	Yes: 500 °C	–	retention of nanopores	–
2006	Xu *et al.* Gaithersburg, MD	Directly injectable calcium orthophosphate cement, self hardens, mannitol as porogen.	Nanocrystalline HA	No	2.2–4.2 MPa (flexural)	0–50% macroporous	65–82%
2004	Landi *et al.* Italy + Indonesia	Sponge impregnation, isotactic pressing, sintering of HA in simulated body fluid.	Calcium hydroxide + orthophosphoric acid	Yes: 1250 °C for 1 hr	23 ± 3.8 MPa	closed 6%, open 60%	66%
2003	Charriere *et al.* EPFL, Switzerland	Thermoplastic negative porosity by ink jet printing, slip casting process for HA	DCPD + Calcite	No: 90 °C for 1 day	12.5 ± 4.6 MPa	–	44%
2003	Almirall *et al.* Barcelona, Spain	α-TCP foamed with hydrogen peroxide at different conc., liq. ratios, poured in PTFE molds.	α-TCP + (10% and 20% peroxide)	No: 60 °C for 2 hr	1.41 ± 0.27 MPa 2.69 ± 0.91 MPa	35.7% macro 29.7% micro 26.8% macro 33.8% micro	65.5% 60.7%
2003	Ramay *et al.* Seattle, WA	Slurries of HA prepared: gel-casting + polymer sponge technique. Sintered.	HA powder	Yes: 600 °C for 1 hr, 1350 °C for 2 hr	0.5–5 MPa	200–400 μm	70–77%
2003	Miao *et al.* Singapore	TTCP to calcium orthophosphate cement. Slurry cast on polymer foam, sintered.	TTCP	Yes: 1200 °C for 2 hr	–	1 mm macro, 5 μm micro	~70%
2003	Uemura *et al.* China + Japan	Slurry of HA with polyoxyethylenelaurylether (crosslinked) and sintered.	HA powders	Yes: 1200 °C for 3 hr	2.25 MPa (0 wk) 4.92 MPa (12 wks) 11.2 MPa (24 wks)	500 micron 200 μm interconnects	~77%
2003	Ma *et al.* Singapore + USA	Electrophoretic deposition of HA, sintering.	HA powders	Yes: 1200 °C for 2 hr	860 MPa	0.5 μm, 130 μm	~20%
2002	Barralet *et al.* Birmingham, London	Calcium orthophosphate cement + sodium orthophosphate ice: evaporated.	Calcium carbonate + DCDP	1st step: 1400 °C for 1 day	0.6 ± 0.27 MPa	2 μm	62 ± 9%

Porous bioceramics with an improved strength might be fabricated from calcium orthophosphate fibers or whiskers. In general, fibrous porous materials are known to exhibit improved strength due to fiber interlocking, crack deflection and/or pullout [386]. Namely, porous bioceramics with well-controlled open pores were processed by sintering of fibrous HA particles [387]. In another approach, porosity was achieved by firing apatite-fiber compacts mixed with carbon beads and agar. By varying the compaction pressure, firing temperature and carbon/HA ratio, the total porosity was controlled in the ranges from ~40% to ~85% [378]. Additional examples are available in literature [364,367,374,375,376,377,378,379,380,389,390,391,392,393,394,395,396,397,398,399,400,401,402,403,404,405,406,407,408].

*In vivo* response of calcium orthophosphate bioceramics of different porosity was investigated and hardly any effect of macropore dimensions (~150, ~260, ~510 and ~1220 μm) was observed [409]. In another study, a greater differentiation of mesenchymal stem cells was observed when cultured on ~200 μm pore size HA scaffolds when compared to those on ~500 μm pore size HA [410]. The latter finding was attributed to the fact that a higher pore volume in ~500 μm macropore scaffolds might contribute to a lack of cell confluency, leading to the cells proliferating before beginning differentiation. In addition, the authors hypothesized that bioceramics having less than optimal pore dimensions induced quiescence in differentiated osteoblasts due to reduced cell confluency [410]. Already in 1979, Holmes suggested that the optimal pore range was 200–400 μm with the average human osteon size of ~223 μm [105]. In 1997, Tsurga and coworkers implied that the optimal pore size of bioceramics that supported ectopic bone formation was 300–400 μm [411]. Thus, there is no need to create calcium orthophosphate bioceramics with very big pores; however, the pores must be interconnected [108,352,364,365]. Interconnectivity governs a depth of cells or tissue penetration into the porous bioceramics, as well as allowing development of blood vessels required for new bone nourishing and waste removal [412,413].

**Figure 6:**
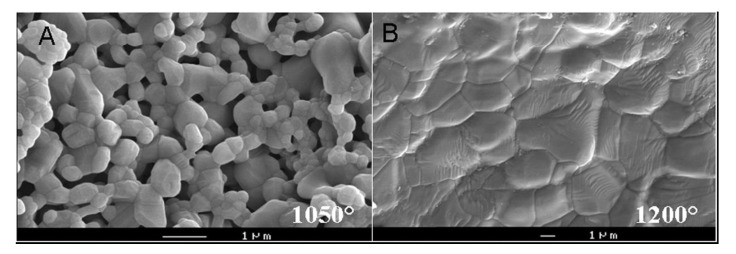
SEM pictures of HA bioceramics sintered at (**a**) 1050 °C and (**b**) 1200 °C. Note the presence of microporosity in (**a**) and not in (**b**). Reprinted from [415] with permission.

Bioceramic microporosity (pore size <10 μm), which is defined by its capacity to be impregnated by biological fluids [412], results from the sintering process, while the pore dimensions mainly depend on the material composition, thermal cycle and sintering time. The microporosity provides both a greater surface area for protein adsorption and increased ionic solubility. Nanoporous (average pore sizes of less than 100 nm) HA bioceramics might be fabricated as well [414]. Differences in porogens influence the macroporosity, while differences in sintering temperatures and conditions affect the percentage of microporosity. Usually, the higher the sintering temperature, the lower both the microporosity content and the specific surface area of bioceramics. Namely, HA bioceramics sintered at ~1200 °C shows significantly less microporosity and a dramatic change in crystal sizes, if compared with those sintered at ~1050 °C (Figure 6). Furthermore, the average shape of pores was found to transform from strongly oblate to round at higher sintering temperatures [416]. The total porosity (macroporosity + microporosity) of calcium orthophosphate bioceramics was reported to be about 70% of the bioceramic volume [417]. In the case of coralline HA or bovine-derived apatites, the porosity of the original biologic material (coral or bovine bone) is usually preserved during processing [107]. To conclude this topic, creation of the desired porosity in calcium orthophosphate bioceramics is a rather complicated engineering task and interested readers are referred to [65,119,368,382,418,419,420,421,422,423,424,425,426,427,428,429,430,431,432,433,434,435,436,437,438,439,440,441,442].

Studies revealed that increasing both the specific surface area and pore volume of bioceramics might greatly accelerate the *in vivo* process of apatite deposition and, therefore, enhance bone-forming bioactivity. More importantly, a precise control over the porosity, pore dimensions and internal pore architecture of bioceramics on different length scales is essential for understanding the structure-bioactivity relationship and the rational design of better bone-forming biomaterials [439,443,444]. Namely, in antibiotic charging experiments, a nanoporous calcium orthophosphate bioceramic showed a much higher charging capacity (1621 μg/g) than that of commercially available calcium orthophosphate (100 μg/g), which did not have any nanoporosity [434]. In other experiments, porous blocks of HA were found to be viable carriers with sustained release profiles for drugs [445] and antibiotics over 12 days [446] and 12 weeks [447], respectively. Unfortunately, the porosity significantly decreases the strength of implants [63,283,301]. Thus, porous calcium orthophosphate implants cannot be loaded and are used to fill only small bone defects. However, their strength increases gradually when bones ingrow into the porous network of calcium orthophosphate implants [448,449,450,451]. For example, Martin *et al.* reported bending strengths of 40–60 MPa for a porous HA implant filled with 50–60% of cortical bone [448], while in another study an ingrown bone increased strength of porous HA bioceramics by a factor of three to four [450].

To conclude this topic, filters for microbial filtration might be manufactured from porous HA [452].

## 6. Biomedical Applications

Since Levitt *et al.* described a method of preparing a FA bioceramics and suggested their possible use in medical applications in 1969 [453], calcium orthophosphate bioceramics have been widely tested for clinical applications. Namely, calcium orthophosphates in a number of forms and compositions (Table 3) are currently either in use or under a consideration in many areas of dentistry and orthopedics, with even more in development. For example, bulk materials, available in dense and porous forms, are used for alveolar ridge augmentation, immediate tooth replacement and maxillofacial reconstruction [65,69]. Other examples include orbital implants (Bio-Eye^®^) [458,459], increment of the hearing ossicles, spine fusion and repair of bone defects [460,461]. In order to permit growth of new bone into defects, a suitable bioresorbable material should fill these defects. Otherwise, ingrowth of fibrous tissue might prevent bone formation within the defects.

**Table 3:** Various examples of the commercially available calcium orthophosphate-based bioceramics and biomaterials [12,415,417,454,455,456,457].

Calcium orthophosphate		Trade name and producer	
CDHA		Cementek (Teknimed, France)	
		Osteogen (Impladent, NY, USA)	
HA		Actifuse (ApaTech, UK)	
		Apaceram (Pentax, Japan)	
		ApaPore (ApaTech, UK)	
		Bioroc (Depuy-Bioland, France)	
		Bonefil (Pentax, Japan)	
		Bonetite (Pentax, Japan)	
		Boneceram (Sumitomo Osaka Cement, Japan)	
		BoneSource (Stryker Orthopaedics, NJ, USA)	
		Calcitite (Zimmer, IN, USA)	
		Cerapatite (Ceraver, France)	
		Neobone (Toshiba Ceramics, Japan)	
		Ostegraf (Ceramed, CO, USA)	
		Ostim (Heraeus Kulzer, Germany)	
		Synatite (SBM, France)	
HA/collagen		Bioimplant (Connectbiopharm, Russia)	
		Bonject (Koken, Japan)	
		CollapAn (Intermedapatite, Russia)	
		HAPCOL (Polystom, Russia)	
		LitAr (LitAr, Russia)	
HA/sodium alginate		Bialgin (Biomed, Russia)	
HA/Poly-L-Lactic Acid		SuperFIXSORB30 (Takiron, Japan)	
HA/polyethylene		HAPEX (Gyrus, TN, USA)	
HA/CaSO_4_		Hapset (LifeCore, MIN, USA)	
coralline HA		Interpore (Interpore, CA, USA)	
		ProOsteon (Interpore, CA, USA)	
algae-derived HA		Algipore (Dentsply Friadent, Germany)	
bovine bone apatite (unsintered)		BioOss (Geitslich, Switzerland)	
		Laddec (Ost-Developpement, France)	
		Lubboc (Ost-Developpement, France)	
		Oxbone (Bioland biomateriaux, France)	
		Tutoplast (IOP, CA, USA)	
bovine bone apatite (sintered)		BonAP	
		Cerabone (aap Implantate, Germany)	
		Endobon (Merck, Germany)	
		Osteograf (Ceramed, CO, USA)	
		PepGen P-15 (Dentsply Friadent, Germany)	
β-TCP		Bioresorb (Sybron Implant Solutions, Germany)	
		Biosorb (SBM S.A., France)	
		Calciresorb (Ceraver, France)	
		Cerasorb (Curasan, Germany)	
		Ceros (Thommen Medical, Switzerland)	
		ChronOS (Synthes, PA, USA)	
		Conduit (DePuy Spine, USA)	
		JAX (Smith and Nephew Orthopaedics, USA)	
		Osferion (Olympus Terumo Biomaterials, Japan)	
		OsSatura TCP (Integra Orthobiologics, CA, USA)	
		Vitoss (Orthovita, PA, USA)	
BCP (HA + β-TCP)		4Bone (MIS, Israel)	
		BCP (Medtronic, MN, USA)	
		Biosel (Depuy Bioland, France)	
		BoneSave (Stryker Orthopaedics, NJ, USA)	
		Calciresorb (Ceraver, France)	
		CellCeram (Scaffdex, Finland)	
		Ceraform (Teknimed, France)	
		Ceratite (NGK Spark Plug, Japan)	
		Eurocer (FH Orthopedics, France)	
		Graftys BCP (Graftys, France)	
		Hatric (Arthrex, Naples, FL, USA)	
		Indost (Polystom, Russia)	
		Kainos (Signus, Germany)	
		MBCP (Biomatlante, France)	
		OptiMX (Exactech, USA)	
		OsSatura BCP (Integra Orthobiologics, CA, USA)	
		Osteosynt (Einco, Brazil)	
		SBS (Expanscience, France)	
		TCH (Kasios, France)	
		Triosite (Zimmer, IN, USA)	
		Tribone (Stryker, Europe)	
BCP (HA + α-TCP)		Skelite (Millennium Biologix, ON, Canada)	
BCP/collagen		Allograft (Zimmer, IN, USA)	
BCP/fibrin		TricOS (Baxter BioScience, France)	
BCP/silicon		FlexHA (Xomed, FL, USA)	
FA + BCP (HA + β-TCP)		FtAP (Polystom, Russia)	
carbonateapatite		Healos (Orquest, CA, USA)	

In spite of the aforementioned serious mechanical limitations, bioceramics of calcium orthophosphates are available in various physical forms: powders, particles, granules (or granulates [11]), dense blocks, porous scaffolds, injectable formulations, self-setting cements and concretes, implant coatings and composite component of different origin (natural, biological or synthetic) often with specific shapes, such as implants, prostheses or prosthetic devices (Table 4) [1,108]. Furthermore, bone grafts are also proposed as non-hardening pastes (=“putty”). Generally, the latter materials consist of a mixture of calcium orthophosphate granules and a “glue”, typically a highly viscous hydrogel [1,292]. More to the point, custom-designed shapes like wedges for tibial opening osteotomy, cones for spine and knee and inserts for vertebral cage fusion are also available [417]. Various trademarks of the commercially available types of calcium orthophosphate-based bioceramics and biomaterials are summarized in Table 3.

### 6.1. Cements and Concretes

The need of bioceramics for minimal invasive surgery has induced the development of a concept of self-setting bone cements consisting of only calcium orthophosphates to be applied as injectable and/or mouldable bone substitutes [149,366,367,384,431,462,463,464,465,466,467,468,469,470]. In addition, there are reinforced formulations, which, in a certain sense, might be defined as calcium orthophosphate concretes [464]. Furthermore, porous formulations of both the cements and the concretes are available [367,384,465,466,467,468].

**Figure 7:**
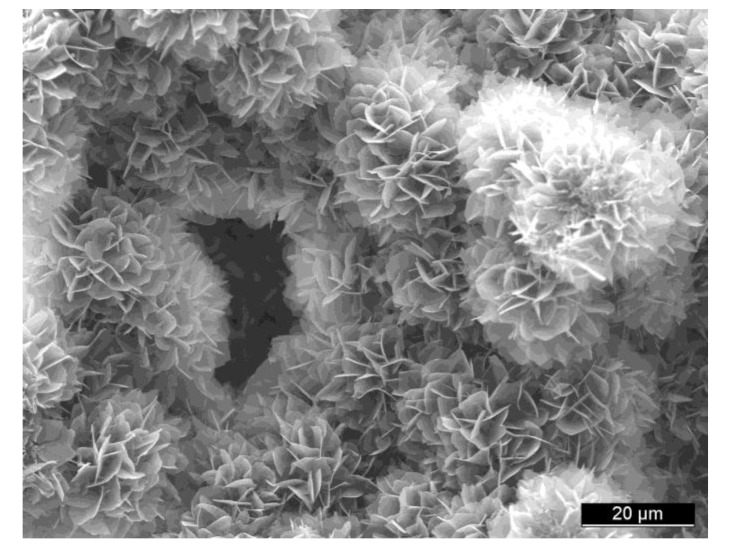
A typical microstructure of a calcium orthophosphate cement after hardening. The mechanical stability is provided by the physical entanglement of crystals. Reprinted from [1] with permission.

Calcium orthophosphate cements and concretes belong to low temperature bioceramics. They are divided into two major groups. The first one is a dry mixture of two different calcium orthophosphates (a basic one and an acidic one), in which, after being wetted, the setting reaction occurs according to an acid-base reaction. The second group of the cements contains only one calcium orthophosphate. Typical examples include ACP with Ca/P molar ratio within 1.50–1.67 and α-TCP: they form CDHA upon contact with an aqueous solution [149,463,464]. The setting reaction (= hardening, curing) of these materials is initiated by mixing the initial powder(s) with an aqueous solution. Chemically, hardening is due to the successive dissolution and precipitation reactions. Mechanically, hardening results from crystal entanglement and intergrowth (Figure 7) [1]. Setting of calcium orthophosphate cements and concretes occurs mostly within the initial ~6 h, yielding ~80% conversion to the final products and a compressive strength of 40–60 MPa. Hardening rate is strongly influenced by powder to liquid ratio, as well as by addition of other chemicals [149,462,463,464,465,466,467,468,469,470]. Despite a large number of formulations, all calcium orthophosphate cements can only form two different end products: CDHA and DCPD [149,463,464].

All calcium orthophosphate cements and concretes are biocompatible, bioactive and bioresorbable. The first animal study of a calcium orthophosphate cement was performed in 1991, where a cement consisting of TTCP and DCPA was investigated histologically by implanting disks made of this cement within the heads of nine cats [471,472]. In 1996, that formulation received an approval by the U.S. Food and Drug Administration, thus becoming the first commercially available calcium orthophosphate cement for use in humans [465]. As the structure and composition of the hardened cements is close to that of bone mineral, the material of the hardened cements can easily be used by bone remodeling cells for reconstruction of damaged parts of bones [149,462,463,464,465]. A possibility to be injected (a minimally invasive technique), a low setting temperature, an adequate stiffness, an easy shaping and a good adaptation to the defect geometry are the major advantages of calcium orthophosphate cements and concretes, when compared to the prefabricated bulk bioceramics and porous scaffolds. Further details on this subject are available in literature [463,464,465].

**Table 4:** Specific features of the four most common forms of bone graft substitutes. The column “defect form” lists the types of defects that can be potentially filled with the listed bone graft substitute form. “Open” means that the defect has to be widely open, e.g., an open cancellous bone defect; “Defined shape” means that the defect has to have a well-defined shape, e.g., cylinder; “Closed” means that the material can be (potentially) injected into a closed defect, e.g., to reinforce an osteoporotic bone [1].

Form	Defect form	Mechanical stability	Resorption/bone formation	Handling
Granules (0.1–5 mm in diameter)	Open	Negligible	Throughout the defect	Fair (granule migration during and after surgery)
Macroporous blocks	Open and defined shape	Fair provided there is press-fitting into the defect	Throughout the defect	Very good (problems might arise to fit the block within the defect)
Cement paste	Closed	Fair	Peripheral	Fair to good (the paste might set too fast or might be poorly injectable)
Putty	Open or closed	Negligible	Peripheral or throughout the defect depending on the composition	Very good for pastes that have to be mixed in the operating room to excellent for ready-mixed pastes (the paste might be poorly-injectable)

### 6.2. Coatings

For many years, the clinical application of calcium orthophosphate-based bioceramics has been largely limited to non-load bearing parts of the skeleton due to their inferior mechanical properties. One of the major innovations in the last ~30 years has been to coat mechanically strong bioinert and/or biotolerant prostheses by calcium orthophosphates [60,473,474]. For example, metallic implants are encountered in endoprostheses (total hip joint replacements) and artificial teeth sockets. The requirement for a sufficient mechanical stability necessitates the use of a metallic body for such devices. As metals do not undergo bone bonding, *i.e*., do not form a mechanically stable link between the implant and bone tissue, methods have been sought to improve contacts at the interface. The major way is to coat metals with calcium orthophosphate bioceramics that exhibit a bone-bonding ability between the metal and bone [60,179,190,315,475,476,477,478,479,480]. Thickness of the coatings vary from submicron dimensions to several hundreds microns (Table 5) and this parameter appears to be very important. For example, if a calcium orthophosphate coating is too thick, it is easy to break. On the contrary, if the coating is too thin, it is easy to dissolve, because resorbability of HA, which is the second slowest to dissolve among calcium orthophosphates (Table 1), is about 15–30 μm per year [481]. One should stress that calcium orthophosphate coatings are not limited to metals only; they can be applied on carbon, bioinert ceramics and polymers as well [482]. Most important coating techniques are listed in Table 5, while the main advantages and drawbacks of each coating technique, as well as the major properties of the deposed calcium orthophosphates, are discussed in detail elsewhere [60,179,221,272,473,483,484,485,486,487,488,489,490,491,492,493,494,495,496,497]. Unfortunately, none of these methods can provide the perfect covering because each coating always contains cracks, pores, second phases and residual stresses that reduced their durability and might lead to a partial or complete disintegration of the coating in body fluids. The biomedical aspects of osteoconductive coatings for total joint arthroplasty have been reviewed elsewhere [498].

**Table 5:** Various techniques to deposit bioresorbable coatings of calcium orthophosphates on metal implants [484,485].

Technique	Thickness	Advantages	Disadvantages
Thermal spraying	30–200 μm	High deposition rates; low cost	Line of sight technique; high temperatures induce decomposition; rapid cooling produces amorphous coatings
Sputter coating	0.5–3 μm	Uniform coating thickness on flat substrates; dense coating	Line of sight technique; expensive; time consuming; produces amorphous coatings
Pulsed laser deposition	0.05–5 μm	Coating by crystalline and amorphous phases; dense and porous coating	Line of sight technique
Dynamic mixing method	0.05–1.3 μm	High adhesive strength	Line of sight technique; expensive; produces amorphous coatings
Dip coating	0.05–0.5 mm	Inexpensive; coatings applied quickly; can coat complex substrates	Requires high sintering temperatures; thermal expansion mismatch
Sol-gel technique	<1 μm	Can coat complex shapes; low processing temperatures; relatively cheap as coatings are very thin	Some processes require controlled atmosphere processing; expensive raw materials
Electrophoreticdeposition	0.1–2.0 mm	Uniform coating thickness; rapid deposition rates; can coat complex substrates	Difficult to produce crack-free coatings; requires high sintering temperatures
Biomimetic coating	<30 μm	Low processing temperatures; can form bonelike apatite; can coat complex shapes; can incorporate bone growth stimulating factors	Time consuming; requires replenishment and a pH constancy of simulated body fluid
Hot isostatic pressing	0.2–2.0 μm	Produces dense coatings	Cannot coat complex substrates; high temperature required; thermal expansion mismatch; elastic property differences; expensive; removal/interaction of encapsulation material
Electrochemical deposition	0.05–0.5 mm	Uniform coating thickness; rapid deposition rates; can coat complex substrates; moderate temperature, low cost	Thecoating/substrate bonding is not strong enough

All coatings must adhere satisfactorily to the underlying substrate irrespective of their intended function. Specifically, mechanical stability of calcium orthophosphate coatings should be high enough to maintain their bioactive functionality after a surgical implantation. Generally, tensile adhesion testing according to standards such as ASTM C633 is the most common procedure to determine the quantitative values for calcium orthophosphate coating adhesion to the underlying metallic substrates. Furthermore, fatigue [499,500] scratch [501] and pullout [501] testing are among the most valuable techniques to provide additional information on the mechanical behavior of calcium orthophosphate coatings [179].

**Figure 8:**
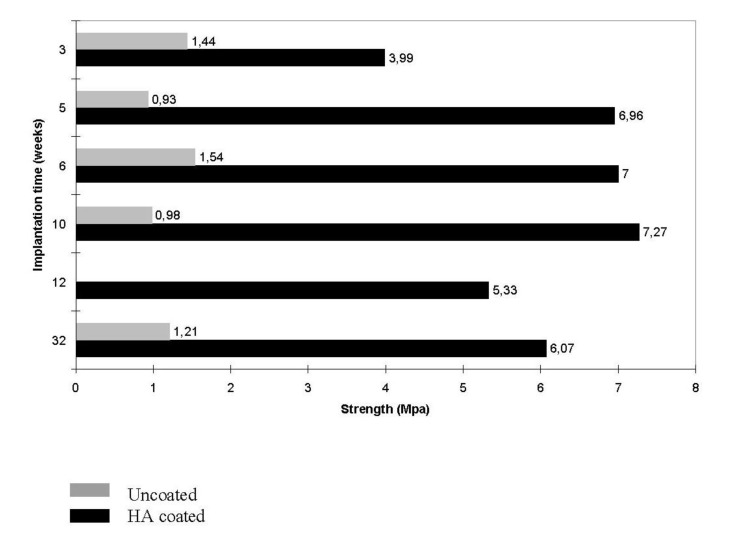
Plasma-sprayed HA coating on a porous titanium (dark bars) is dependent on the implantation time and will improve the interfacial bond strength compared to uncoated porous titanium (light bars). Reprinted from [46] with permission.

Already in the 1980s, de Groot *et al.* [502] published on the development of plasma-sprayed HA coatings on metallic implants. A little bit later, Furlong and Osborn [503], two leading surgeons in the orthopedics field, began implanting plasma-sprayed HA stems in patients. Coated implants combine the surface biocompatibility and bioactivity of calcium orthophosphates with the core strength of strong substrates (Figure 8). Moreover, calcium orthophosphate coatings decrease a release of potentially hazardous chemicals from the core implant and shield the substrate surface from environmental attack. In the case of porous implants, calcium orthophosphate coatings enhance bone ingrowth into the pores [63]. Clinical results for calcium orthophosphate-coated implants reveal that they have much longer life times after implantation than uncoated devices and they are found to be particularly beneficial for younger patients. Studies concluded that there was significantly less pin loosening in the HA-coated groups [504]. HA coating as a system of fixation of hip implants was found to work well in the short to medium term (eight years [505], 10 to 15.5 years [506], 15 years [507], 16 years [508], 17 years [509], 19 years [510] and 15 to 21 years [511]). Similar data for HA-coated dental implants are also available [512,513]. Even longer-term clinical results are awaited with great interest.

A number of factors appear to influence the properties of calcium orthophosphate coatings, including coating thickness (this will influence coating adhesion and fixation—the agreed optimum now seems to be within 50–100 µm), crystallinity (affecting the dissolution and biological behavior), phase purity, chemical purity, porosity and adhesion [473,484]. Methods for the production of coatings and their properties are now largely standardized and, over recent years, calcium orthophosphate-coated implants have found highly successful clinical application, particularly in younger patients [514,515,516]. Further details on calcium orthophosphate coatings can be found in excellent reviews [517,518].

### 6.3. Functionally Graded Bioceramics

In general, functionally gradient materials (FGMs) are defined as materials having either a gradient of compositional or structural changes from their surface to the interior. The idea of FGMs allows one device to possess two different properties. One of the most important combinations for the biomedical field is that of mechanical strength and biocompatibility. Namely, only surface properties govern biocompatibility of the entire device. In contrast, the strongest material determines the mechanical strength of the entire device. Although, this subject belongs to the coatings section (above), in a certain sense, metallic implants covered by calcium orthophosphates can be considered as a FGM. The surface shows excellent biocompatibility because it consists of calcium orthophosphates, while the metallic core provides excellent mechanical strength. The gradient change from calcium orthophosphate to metal is important, for example, from the point of thermal expansion.

**Figure 9:**
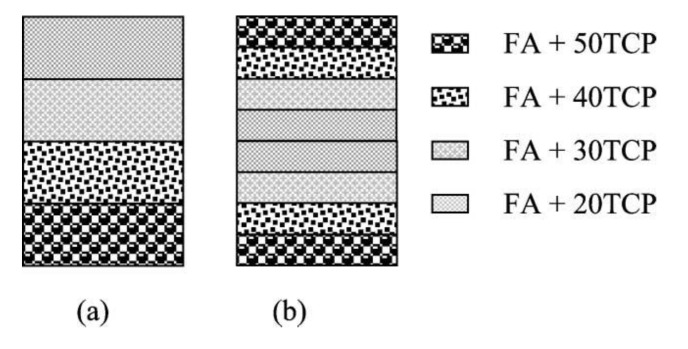
A schematic diagram showing the arrangement of the FA/β-TCP biocomposite layers. (**a**) A non-symmetric functionally gradient material (FGM); (**b**) symmetric FGM. Reprinted from [523] with permission.

Functionally graded bioceramics consisting of calcium orthophosphates only [519] have been developed [377,438,440,522,523,524,525,526,527,528,529]. For example, dense sintered bodies with gradual compositional changes from α-TCP to HA were prepared by sintering diamond-coated HA compacts at 1280 °C under a reduced pressure, followed by heating under atmospheric conditions [522]. The content of α-TCP gradually decreased, while the content of HA increased with increasing depth from the surface. This functionally gradient bioceramic consisting of an HA core and α-TCP surface showed potential value as bone-substituting biomaterial [522]. Two types of functionally gradient FA/β-TCP biocomposites were prepared in another study [523]. As shown in Figure 9, one of the graded biocomposites was in the shape of a disk and contained four different layers of about 1 mm thickness. The other graded biocomposite was also in the shape of a disk but contained two sets of four layers, each layer being 0.5 mm thick, controlled by using a certain amount of the mixed powders. The final FA/β-TCP graded structures were formed at 100 MPa and sintered at 1300 °C for 2 h [523].

Furthermore, it is known that the bone cross-section from cancellous to cortical bone is non-uniform in porosity and pore dimensions. Thus, in various attempts to mimic the porous structure of bones, calcium orthophosphate bioceramics with graded porosity have been fabricated [377,438,440,522,523,524,525,526,527,528,529]. Since diverse biomedical applications require different configurations and shapes, the graded (or gradient) porous bioceramics can be grouped according to both the overall shape and the structural configuration [346]. The basic shapes include rectangular blocks and cylinders (or disks). For the cylindrical shape, there are configurations of dense core-porous layer, less porous core-more porous layer, dense layer-porous core and less porous layer-more porous core. For the rectangular shape, in the gradient direction *i.e*., the direction with varying porosity, pore size or composition, there are configurations of porous top-dense bottom (same as porous bottom-dense top), porous top-dense center-porous bottom, dense top-porous center-dense bottom, *etc*. Concerning biomedical applications, a dense core-porous layer structure is suitable for implants of a high mechanical strength and with bone ingrowth for stabilization, whereas a less porous layer-more porous core configuration can be used for drug delivery systems. Furthermore, a porous top-dense bottom structure can be shaped into implants of articulate surfaces for wear resistance and with porous ends for bone ingrowth fixation; while a dense top-porous center-dense bottom arrangement mimics the structure of head skull. Further details on bioceramics with graded porosity might be found in literature [346].

## 7. Biological Properties and In Vivo Behavior

The most important differences between bioactive bioceramics and all other implanted materials are: inclusion in metabolic processes of the organism; adaptation of either surface or the entire material to the biomedium; integration of a bioactive implant with bone tissues at the molecular level or the complete replacement of a resorbable bioceramic by healthy bone tissues. All of the enumerated processes are related to the effect of an organism on the implant. Nevertheless, another aspect of implantation is also important—the effect of the implant on the organism. For example, use of bone implants from corpses or animals, even after they have been treated in various ways, provokes a negative immune reaction in the organism, which substantially limits the application of such implants. In this connection, it is useful to dwell on the biological properties of bioceramic implants, particularly those of calcium orthophosphates, which in the course of time may be resorbed completely [530].

### 7.1. Interaction with the Surrounding Tissues and the Host Responses

An interaction between an implant and surrounding tissues is a dynamic process. Water, dissolved ions, biomolecules and cells surround the implant surface during initial few seconds after the implantation. It is accepted that no foreign material placed within a living body is completely compatible. The only substances that conform completely are those manufactured by the body itself (autogenous) and any other substance that is recognized as foreign, initiates some reactions (a host-tissue response). The reactions occurring at the biomaterial/tissue interface lead to time-dependent changes in the surface characteristics of both the implanted biomaterials and the surrounding tissues [54,531].

In order to develop new biomaterials, it is necessary to understand the *in vivo* host responses. Like any other species, biomaterials and bioceramics react chemically with their environment and, ideally, they should neither induce any changes nor provoke undesired reactions in the neighboring or distant tissues. In general, living organisms can treat artificial implants as biotoxic (or bioincompatible [50]), bioinert (or biostable [42]), biotolerant (or biocompatible [50]), bioactive and bioresorbable materials [3,4,5,6,38,39,40,46,47,48,49,50,91,478,530,531,532]. Biotoxic (e.g., alloys containing cadmium, vanadium, lead and other toxic elements) materials release substances to the body in toxic concentrations and/or trigger the formation of antigens that may cause immune reactions ranging from simple allergies to inflammation to septic rejection with the associated severe health consequences. They cause atrophy, pathological change or rejection of living tissue near the material due to chemical, galvanic or other processes. Bioinert [533] (e.g., zirconia, alumina, carbon and titanium) and biotolerant (e.g., polymethylmethacrylate, titanium and Co-Cr alloy) materials do not release any toxic constituents but also do not show positive interaction with living tissue. They evoke a physiological response to form a fibrous capsule, thus, isolating the material from the body. In such cases, thickness of the layer of fibrous tissue separating the material from other tissues of an organism can serve as a measure of bioinertness. Generally, both bioactivity and bioresorbability phenomena are fine examples of chemical reactivity and calcium orthophosphates (both non-substituted and ion-substituted ones) fall into these two categories of bioceramics [3,4,5,6,38,39,40,46,47,48,49,50,91,478,530,531,532]. A bioactive material will dissolve slightly but promote formation of a surface layer of biological apatite before interfacing directly with the tissue at the atomic level, which results in formation of a direct chemical bonds to bones. Such an implant provides good stabilization for materials that are subject to mechanical loading. A bioresorbable material will dissolve over time (regardless of the mechanism leading to the material removal) and allow a newly formed tissue to grow into any surface irregularities but may not necessarily interface directly with the material. Consequently, the functions of bioresorbable materials are to participate in dynamic processes of formation and re-absorption occurring in bone tissues; thus, bioresorbable materials are used as scaffolds or filling spacers allowing their infiltration and substitution to the tissues [47,272,534,535,536,537].

A distinction between bioactive and bioresorbable bioceramics might be associated with a structural factor only. For example, bioceramics made from non-porous, dense and highly crystalline HA behaves as a bioinert (but a bioactive) material and are retained in an organism for at least 5–7 years without noticeable changes, while a highly porous bioceramics of the same composition can be resorbed approximately within a year. Furthermore, submicron-sized HA powders are biodegraded even faster than the highly porous HA scaffolds. Other examples of bioresorbable materials include porous bioceramic scaffolds made of BCP (which is an intimate mixture of either β-TCP + HA [118,119,120,121,122,123,124,125,126,127,128,129,130], or α-TCP + HA [7,8,9,10,11]) or bone grafts (dense or porous) made of CDHA [538], TCP [377,539,540] and/or ACP [418,541]. One must stress that recently the concepts of bioactive and bioresorbable materials have converged and bioactive materials are made bioresorbable, while bioresorbable materials are made bioactive [542].

In certain *in vivo* experiments an inflammatory reaction was observed after implantation of calcium orthophosphate bioceramics [543,544,545]. Despite this, the general conclusion on using calcium orthophosphates with a Ca/P ionic ratio within 1.0–1.7 is that all types of implants (bioceramics of various porosities and structures, powders or granules) are not only nontoxic but also induce neither inflammatory nor foreign-body reactions [546]. The biological response to implanted calcium orthophosphates follows a similar cascade observed in fracture healing. This cascade includes hematoma formation, inflammation, neovascularization, osteoclastic resorption and new bone formation. An intermediate layer of fibrous tissue between the implants and bones has never been detected. Furthermore, calcium orthophosphate implants display the ability to directly bond to bones [2,3,4,5,6,38,42,46,47,48,49,50,51,52,54,60,530]. For further details, interested readers are referred to a good review on cellular perspectives of bioceramic scaffolds for bone tissue engineering [374].

One should note that the aforementioned rare cases of inflammatory reactions to calcium orthophosphate bioceramics were caused by “other” reasons. For example, a high rate of wound inflammation occurred when highly porous HA was used. In that particular case, the inflammation was explained by sharp implant edges, which irritated surrounding soft tissues [544]. Another reason for inflammation produced by porous HA could be due to micro movements of the implants, leading to simultaneous disruption of a large number of micro-vessels, which grow into the pores of the bioceramics. This would immediately produce an inflammatory reaction. Additionally, problems could arise in clinical tests connected with migration of granules used for alveolar ridge augmentation, because it might be difficult to achieve mechanical stability of implants at the implantation sites [544].

### 7.2. Osteoinduction

Until recently, it was generally considered, that alone, any type of synthetic bioceramics possessed neither osteogenic [547] nor osteoinductive [548] properties and demonstrated minimal immediate structural support. When attached to healthy bones, an osteoid [550] is produced directly onto the surfaces of bioceramics in the absence of a soft tissue interface. Consequently, the osteoid is mineralized and the resulting new bone undergoes remodeling [549]. However, several reports have already shown osteoinductive properties of certain types of calcium orthophosphate bioceramics [168,415,457,551,552,553,554,555,556,557,558,559,560,561,562,563]. Namely, bone formation was found to occur in dog muscle inside porous calcium orthophosphates with surface microporosity, while bone was not observed on the surface of dense bioceramics [559]. Furthermore, implantation of porous β-TCP bioceramics appeared to induce bone formation in soft tissues of dogs, while no bone formation was detected in any α-TCP implants [556]. More to the point, titanium implants coated by a microporous layer of OCP were found to induce ectopic bone formation in goat muscles, while a smooth layer of carbonated apatite on the same implants was not able to induce bone formation there [562,563].

Although the mechanisms of intrinsic osteoinduction of calcium orthophosphate bioceramics are not unraveled, the dissolution/precipitation behavior of calcium orthophosphates [479], as well as their microporosity [564,565] and specific surface area [565] have been pointed out as the relevant parameters. A positive effect of increased microporosity on ectopic bone formation could be direct and indirect. Firstly, an increased microporosity is directly related to the changes in surface topography, *i.e*., increases a surface roughness, which might affect cellular differentiation. Secondly, an increased microporosity indirectly means a larger surface is exposed to the body fluids, leading to elevated dissolution/precipitation phenomena as compared to non-microporous surfaces. Furthermore, other hypotheses are available. Namely, Reddi explained the apparent osteoinductive properties as an ability of particular bioceramics to concentrate bone growth factors, which are circulating in biological fluids, and that these growth factors induce bone formation [566]. Other researchers proposed a similar hypothesis that the intrinsic osteoinduction by calcium orthophosphate bioceramics is a result of adsorption of osteoinductive substances on their surface [561]. Moreover, Ripamonti [567] and Kuboki *et al.* [568] independently postulated that the geometry of calcium orthophosphate bioceramics is a critical parameter in bone induction. Specifically, bone induction by calcium orthophosphates was never observed on flat bioceramic surfaces. All osteoinductive cases were observed on either porous structures or structures contained well-defined concavities. Moreover, bone formation was never observed on the peripheries of porous implants and was always found inside the pores or concavities, aligning the surface [179]. Some researchers speculated that a low oxygen tension in the central region of implants might provoke a dedifferentiation of pericytes from blood micro-vessels into osteoblasts [569]. Finally, and importantly, both nano-structured rough surfaces and a surface charge on implants were found to cause an asymmetrical division of the stem cells into osteoblasts, which is important for osteoinduction [564].

### 7.3. Biodegradation

Shortly after implantation, a healing process is initiated by compositional changes of the surrounding bio-fluids and adsorption of biomolecules. Following this, various types of cells reach the bioceramic surface and the adsorbed layer dictates the ways the cells respond. Further, a biodegradation of the implanted bioceramics begins. This process can occur by either physicochemical dissolution with a possibility of phase transformation or cellular activity (so called, bioresorption), as well as by a combination of both processes. Dissolution is a physical chemistry process, which is controlled by some factors, such as solubility of the implant matrix (Table 1), surface area to volume ratio, local acidity, fluid convection and temperature. For HA, the dissolution process in acids has been described by a sequence of four successive chemical equations [570,571]:

Ca_10_(PO_4_)_6_(OH)_2_ + 2H^+^ → Ca_10_(PO_4_)_6_(H_2_O)_2_^2+^ (1)

Ca_10_(PO_4_)_6_(H_2_O)_2_^2+^ → 3Ca_3_(PO_4_)_2_ + Ca^2+ ^+ 2H_2_O (2)

Ca_3_(PO_4_)_2_ + 2H^+ ^→ Ca^2+^ + 2CaHPO_4_ (3)

CaHPO_4_ + H^+ ^→ Ca^2+^ + H_2_PO_4_^−^ (4)

With few exceptions, dissolution rates of calcium orthophosphates are inversely proportional to the Ca/P ratio, phase purity and crystalline size, as well as being directly related to the porosity and surface area. Phase transformations might occur with OCP, DCPA, DCPD, α-TCP, β-TCP and ACP because they are unstable in aqueous environments under physiological conditions. Bioresorption is a biological process mediated by cells (mainly osteoclasts and to a lesser extent, macrophages). It depends on the response of cells to their environment. Osteoclasts attach firmly to the implant and dissolve calcium orthophosphates by secreting an enzyme carbonic anhydrase or any other acid, leading to a local pH drop to ~4–5 [572]. Furthermore, calcium orthophosphate particles can also be phagocyotosed by osteoclasts, *i.e*., they are incorporated into the cytoplasm and thereafter dissolved by acid attack and/or enzymatic processes. In any case, biodegradation of calcium orthophosphates is a combination of various non-equilibrium processes, occurring simultaneously and/or in competition with each other.

Usually, an *in vitro* biodegradation of calcium orthophosphate bioceramics is estimated by suspending the material in a slightly acidic (pH ~5) buffer and monitoring the release of Ca^2+^ ions with time. The acidic buffer, to some extent, mimics the acidic environment during osteoclastic activity. One study compared the *in vivo* behavior of porous β-TCP bioceramics prepared from rod-shaped particles and that prepared from non-rod-shaped particles in the rabbit femur. Although the porosities of both types of β-TCP bioceramics were almost the same, more active osteogenesis was preserved in the region where rod-shaped bioceramics was implanted [573]. This result implied that the microstructure affected the activity of bone cells and subsequent bone replacement.

Experimental results demonstrated that both the dissolution kinetics and *in vivo* biodegradation of biologically relevant calcium orthophosphates proceed in the following decreasing order: β-TCP > bovine bone apatite (unsintered) > bovine bone apatite (sintered) > coralline HA > HA. In the case of BCP bioceramics, the biodegradation kinetics depends on the HA/TCP ratio: the higher the ratio, the lower the degradation rate. Similarly, *in vivo* degradation rate of biphasic TCP (BTCP, consisting of α-TCP + β-TCP) bioceramics appeared to be lower than that of α-TCP and higher than that of β-TCP bioceramics, respectively [141]. Furthermore, incorporation of doping ions can either increase (e.g., CO_3_^2−^, Mg^2+^ or Sr^2+^) or decrease (e.g., F^−^) the solubility (therefore, biodegradability) of CDHA and HA. Contrarily to apatites, solubility of β-TCP decreases due to incorporation of either Mg^2+^ or Zn^2+^ ions [415]. One should remember that ion-substituted calcium orthophosphates are not considered in this review; interested readers are advised to [7,8,9,10,11,12,13,14,15,16,17,18,19,20,21,22,23,24,25,26,27,28,29,30,31,32,33,34,35,36,37].

### 7.4. Bioactivity

Generally, bioactive materials interact with surrounding bone resulting in formation of a chemical bond to this tissue (bone bonding). The bioactivity phenomenon is determined by both chemical factors, such as crystal phases and molecular structures of a biomaterial, and physical factors, such as surface roughness and porosity. Currently, it is agreed that the newly formed bone bonds directly to biomaterials through a carbonated CDHA layer precipitating at the bone/biomaterial interface. Strange enough, just a few publications are present in the literature [415,484,574,575] that briefly describe the bioactivity mechanism of calcium orthophosphates. For example, the chemical changes occurring after exposure of a synthetic HA bioceramics to both *in vivo* (implantation in human) and *in vitro* (cell culture) conditions were studied. A small amount of HA was phagocytozed but the major remaining part behaved as a secondary nucleator as evidenced by the appearance of newly formed mineral [574]. *In vivo*, cellular activity (e.g., of macrophages or osteoclasts) associated with an acidic environment were found to result in partial dissolution of calcium orthophosphates, causing liberation of calcium and orthophosphate ions to the microenvironment. The liberated ions increased the local supersaturation degree of the surrounding biologic fluids, causing precipitation of nanocrystals of biological apatite with simultaneous incorporation of various ions presented in the fluids. Infrared spectroscopic analyses demonstrated that these nanocrystals were intimately associated with bioorganic components (probably proteins), which might also have originated from the biologic fluids, such as serum [415].

**Figure 10:**
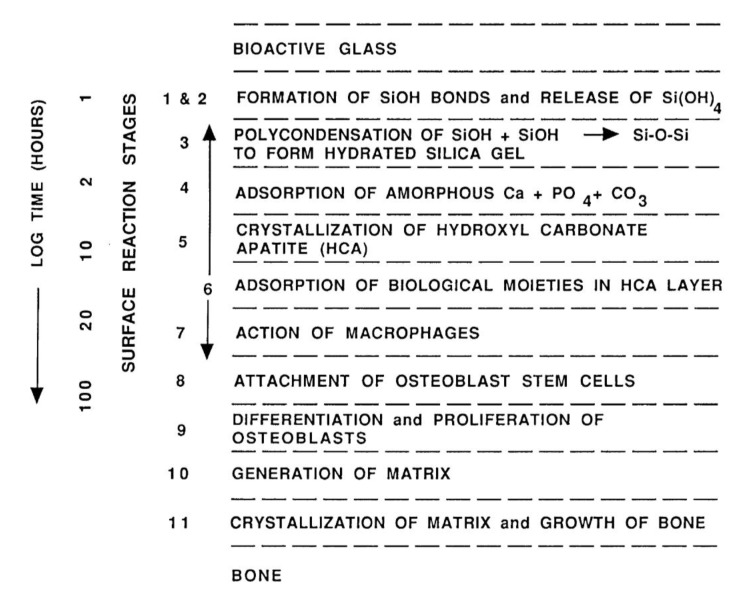
A sequence of interfacial reactions involved in forming a bond between tissue and bioactive ceramics. Reprinted from [46,47,48] with permission.

Therefore, one should better rely on the bioactivity mechanism of other biomaterials, particularly of bioactive glasses—the concept introduced by Larry L. Hench [46,47,48]. The bonding mechanism of bioactive glasses to living tissues involves a sequence of 11 successive reaction steps. The initial five steps occurring on the surface of bioactive glasses are “chemistry” only, while the remaining six steps belong to “biology”; the latter including colonization by osteoblasts, followed by proliferation and differentiation of the cells to form a new bone that had a mechanically strong bond to the implant surface (Figure 10). Therefore, in the case of bioactive glasses, the border between “dead” and “alive” is postulated between stages five and six. According to Hench, all bioactive materials “form a bone-like apatite layer on their surfaces in the living body and bond to bone through this apatite layer. The formation of bone-like apatite on artificial material is induced by functional groups, such as Si-OH (in the case of biological glasses), Ti-OH, Zr-OH, Nb-OH, Ta-OH, -COOH and -H_2_PO_4_ (in the case of other materials). These groups have specific structures revealing negatively charge and induce apatite formation via formations of an amorphous calcium compound, e.g., calcium silicate, calcium titanate and ACP” [46,47,48].

**Figure 11:**
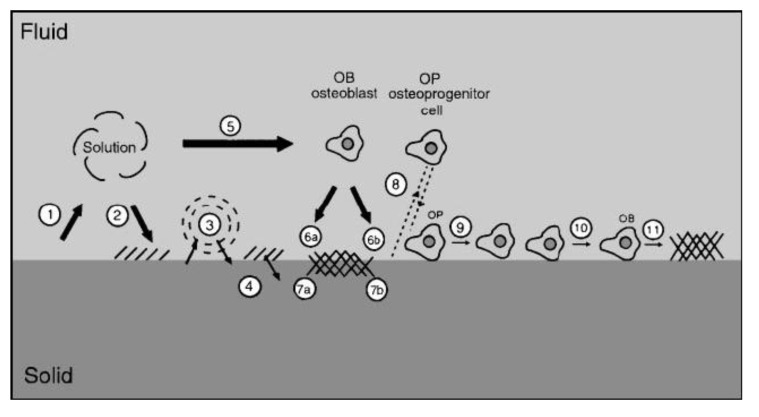
A schematic diagram representing the events taking place at the interface between bioceramics and the surrounding biological environment: (**1**) dissolution of bioceramics; (**2**) precipitation from solution into bioceramics; (**3**) ion exchange and structural rearrangement at the bioceramic/tissue interface; (**4**) interdiffusion from the surface boundary layer into the bioceramics; (**5**) solution-mediated effects on cellular activity; (**6**) deposition of either the mineral phase (a) or the organic phase (b) without integration into the bioceramic surface; (**7**) deposition with integration into the bioceramics; (**8**) chemotaxis to the bioceramic surface; (**9**) cell attachment and proliferation; (**10**) cell differentiation; (**11**) extracellular matrix formation. All phenomena, collectively, lead to the gradual incorporation of a bioceramic implant into developing bone tissue. Reprinted from [54] with permission.

**Figure 12:**
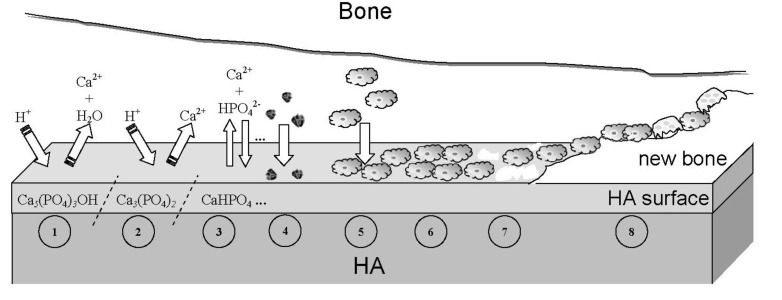
A schematic diagram representing the phenomena that occur on HA surface after implantation: (**1**) beginning of the implant procedure, where solubilization of the HA surface starts; (**2**) continuation of the solubilization of the HA surface; (**3**) the equilibrium between the physiological solutions and the modified surface of HA has been achieved (changes in the surface composition of HA does not mean that a new phase of DCPA or DCPD forms on the surface); (**4**) adsorption of proteins and/or other bioorganic compounds; (**5**) cell adhesion; (**6**) cell proliferation; (**7**) beginning of a new bone formation; (**8**) new bone has been formed. Reprinted from [575] with permission.

To extend the subject, it is important to refer to another set of 11 successive reaction steps for bonding mechanism of unspecified bioceramics, developed by Paul Ducheyne (Figure 11) [54]. One can see that the Ducheyne’s model is rather similar to that proposed by Hench; however, there are noticeable differences. For example, Ducheyne mentions ion exchange and structural rearrangement at the bioceramic/tissue interface (stage 3), as well as interdiffusion from the surface boundary layer into bioceramics (stage 4) and deposition with integration into the bioceramics (stage 7), which are absent in Hench’s model. On the other hand, Hench describes six biological stages (stages 6–11), while Ducheyne describes only four (stages 8–11). Both models were developed almost two decades ago and, to the best of my knowledge, remain unchanged since then. Presumably, both approaches have *pro et contra* of their own and, obviously, should be updated and/or revised. Furthermore, in literature there are at least two other descriptions of biological and cellular events occurring at the bone/implant interface [576,577]; however, they include less stages. One more hypothesis has been proposed recently (Figure 12), which for the first time, describes reasonable surface transformations happening with calcium orthophosphate bioceramics (in that case, HA) shortly after implantation [575].

An important study on formation of calcium orthophosphate precipitates on various types of bioceramic surfaces in both simulated body fluid (SBF) and rabbit muscle sites was performed [578]. The bioceramics were sintered porous solids, including bioglass, glass-ceramics, α-TCP, β-TCP and HA. An ability to induce calcium orthophosphate precipitation was compared among these types of bioceramics. The following conclusions were made: (1) OCP formation ubiquitously occurred on all types of bioceramic surfaces both *in vitro* and *in vivo*, except on β-TCP. (2) Apatite formation did not occur on every type of bioceramic surface; it was less likely to occur on the surfaces of HA and α-TCP. (3) Precipitation of calcium orthophosphates on the bioceramic surfaces was more difficult *in vivo* than *in vitro*. (4) Differences in calcium orthophosphate precipitation among the bioceramic surfaces were less noticeable *in vitro* than *in vivo*. (5) β-TCP bioceramics showed poor calcium orthophosphate precipitation both *in vitro* and *in vivo* [578]. These findings clearly revealed that apatite formation in the physiological environments could not be confirmed to be the common feature of bioceramics. Nevertheless, for want of anything better, currently the bioactivity mechanism of calcium orthophosphate bioceramics could be described by a reasonable combination of Figure 10, Figure 11 and Figure 12, e.g., by updating the Ducheyne’s and Hench’s models by the three initial stages taken from Figure 12.

Interestingly, bioactivity of HA bioceramics might be enhanced by high-energy ion irradiation [579]. The effect was attributed to formation of a unique 3D macroporous apatite layer of decreased crystallinity and crystal size on the irradiated surfaces. To conclude this topic, the atomic and molecular phenomena occurring at the bioceramic surface in aqueous solutions and their effects on the relevant reaction pathways of cells and tissues must be elucidated in more detail. Further investigation of this topic requires a careful analysis of the available experimental data, which is beyond the scope of this review.

### 7.5. Cellular Response

Fixation of an implant in the human body is a dynamic process that remodels the interface zone between the implant and living tissues at all dimensional levels, from the molecular up to the cell and tissue morphology level, and at all time scales, from the first second up to several years after implantation. Immediately following implantation, a space filled with bio-fluids appears next to the implant surface. With time, proteins adsorb at the bioceramic surface and gives rise to osteoinduction by cell proliferation and their differentiation towards bone cells, revascularization and eventual gap closing. Ideally, a strong bond forms between the implant and surrounding tissues [50]. A detailed study on interfacial interactions between calcined HA and substrates has been performed recently [580].

Osteoblasts cultured on HA bioceramics are generally reported to be completely flattened, leading to difficulties in distinguishing the cytoplasmic edge from the HA surfaces after ~2 hours incubation [581]. These observations underscore an expected bioactivity of HA and make HA bioceramics well suited for bone reconstruction. Osteoblasts cultured on porous HA bioceramics appeared to exhibit higher adhesion, enhanced differentiation and suppressed proliferation rates when compared to the non-porous controls [582,583]. Furthermore, formation of distinct resorption pits on HA [584] and β-TCP [573] surfaces in the presence of osteoclasts was observed. Moreover, a surface roughness of calcium orthophosphate bioceramics was reported to strongly influence the activation of mononuclear precursors to mature osteoclasts [584].

Cellular biodegradation of calcium orthophosphate bioceramics is known to depend on its phases. For example, a higher solubility of β-TCP prevented L-929 fibroblast cell adhesion, thereby leading to damage and rupture of the cells [585]. A mouse ectopic model study indicated the maximal bone growth for the 80:20 β-TCP:HA biphasic formulations preloaded with human mesenchymal stem cells when compared to other calcium orthophosphates [586]. The effects of substrate microstructure and crystallinity have been corroborated with an *in vivo* rabbit femur model, where rod-like crystalline β-TCP was reported to enhance osteogenesis when compared to non-rod like crystalline β-TCP [573]. Additionally, using a dog mandibular defect model, a higher bone formation on a scaffold surface coated by nano-dimensional HA was observed when compared to that coated by a micro-dimensional HA [587]. Furthermore, recent studies revealed a stronger stress signaling response by osteoblast precursor cells in 3D scaffolds when compared to 2D surfaces [588].

Mesenchymal stem cells are one of the most attractive cellular lines for application as bone grafts [589]. Early investigations by Okumura *et al.* indicated an adhesion, proliferation and differentiation, which ultimately became new bone and integrated with porous HA bioceramics [590]. Recently, Unger *et al.* showed a sustained co-culture of endothelial cells and osteoblasts on HA scaffolds for up to six weeks [591]. Furthermore, a release of factors by endothelial and osteoblast cells in co-culture supported proliferation and differentiation was suggested to ultimately result in microcapillary-like vessel formation and supported a neo-tissue growth within the scaffold [374]. More to the point, investigation of rat calvaria osteoblasts cultured on transparent HA bioceramics, as well as the analysis of osteogenic-induced human bone marrow stromal cells at different time points of culturing, indicated a good cytocompatibility of HA bioceramics and revealed favorable cell proliferation [343]. Positive results for other types of cells have been obtained in other studies [191,338,339,342].

Interestingly, HA scaffolds with marrow stromal cells in a perfused environment were reported to result in ~85% increase in mean core strength, a ~130% increase in failure energy and a ~355% increase in post-failure strength. The increase in mineral quantity and promotion of uniform mineral distribution in that study was suggested to attribute to the perfusion effect [449]. Additionally, other investigators indicated mechanical properties increased for other calcium orthophosphate scaffolds after induced osteogenesis [448,451].

Furthermore, the dimensions, extent and interconnectivity of pores in bioceramics are known to influence bone in-growth, blood vessels formation and canaliculi networks [409,410,457]. Initial reports have estimated a minimum pore size of ~50 μm for blood vessel formation and a minimum pore size of ~200 μm for osteonal in-growth [457]. Pore dimensions of ~100 μm and even ~50 μm [592] were reported in later studies to support bone in-growth. Additionally, vascularization, cell migration and nutrient diffusion required for sustained cell viability and tissue function are possible if pores within the scaffolds are well interconnected. For example, an essential mean pore interconnection size of ~10 μm was necessary to allow cell migration between the pores [593]. As such, both porosity and general architecture are critical in determining the rate of fluid transport through porous bioceramics, which, in turn, determines the rate and degree of bone ingrowth *in vivo* [122,412,413,594].

## 8. Calcium Orthophosphate Bioceramics in Tissue Engineering

### 8.1. Tissue Engineering

All modern orthopedic implants lack three of the most critical abilities of living tissues: (i) self-repairing; (ii) maintaining blood supply; (iii) self-modifying their structure and properties in response to external aspects such as a mechanical load [429]. Needless to mention, bones not only possess all of these properties but, in addition, are self-generating, hierarchical, multifunctional, nonlinear, composite and biodegradable; therefore, the ideal artificial bone grafts must possess similar properties [117].

The last decades have seen a surge in creative ideas and technologies developed to tackle the problem of repairing or replacing diseased and damaged tissues, leading to the emergence of a new field in healthcare technology now referred to as *tissue engineering*. This is an interdisciplinary field that exploits a combination of living cells, engineering materials and suitable biochemical factors to improve, replace, restore, maintain or enhance living tissues and whole organs [595,596]. However, as two of three major components (namely, cells and biochemical factors) of the tissue engineering subject appear to be far beyond the scope of this review, the topic of tissue engineering is limited to the engineering materials prepared from calcium orthophosphate bioceramics only.

Regeneration, rather than repair, is the central goal of any tissue engineering strategy [597]. Thus, tissue engineering has potential to create tissues and organs *de novo*. This field of science [599] started more than two decades ago [600,601] and a famous review article by Langer and Vacanti [602] has greatly contributed to the promotion of tissue engineering research worldwide. The field of tissue engineering, particularly when applied to bone substitutes where tissues often function in a mechanically demanding environment [603], requires a collaboration of excellence in cell and molecular biology, biochemistry, material sciences, bioengineering and clinical research. For the success, it is necessary that researchers with expertise in one area have an appreciation of the knowledge and challenges of the other areas. However, since the technical, regulatory and commercial challenges might be substantial, the introduction of new products is likely to be slow [598].

Nowadays tissue engineering is at full research potential due to the following key advantages: (i) the solutions it provides are long-term, much safer than other options and cost-effective as well; (ii) the need for a donor tissue is minimal, which eliminates the immuno-suppression problems; (iii) the presence of residual foreign material is eliminated as well.

### 8.2. Scaffolds and Their Properties

It would be very convenient to both patients and physicians if devastated tissues or organs of patients could be regenerated by simple cell injections to the target sites, but such cases are rare. The majority of large-sized tissues and organs with distinct 3D form require a support for their formation from cells. The support is named a scaffold [604], template and/or artificial extracellular matrix (ECM) [151,152,386,600,603,605,606,607,608]. The major function of scaffolds is similar to that of the natural ECM that assists proliferation, differentiation and biosynthesis of cells. In addition, scaffolds placed at the regeneration sites will prevent disturbing cells from invasion into the sites of action [609,610]. The role of scaffolds was perfectly described by Andrés Segovia (1893–1987), a Spanish classical guitarist: “When one puts up a building one makes an elaborate scaffold to get everything into its proper place. But when one takes the scaffold down, the building must stand by itself with no trace of the means by which it was erected. That is how a musician should work.”

The idea behind tissue engineering is to create or engineer autografts by either expanding autologous cells *in vitro* guided by a scaffold or implanting an acellular template *in vivo* and allowing the patient’s cells to repair the tissue guided by the scaffold. The first phase is the *in vitro* formation of a tissue construct by placing the chosen cells and scaffolds in a metabolically and mechanically supportive environment with growth media (in a bioreactor), in which the cells proliferate and elaborate extracellular matrix. It is expected that cells infiltrate into the porous matrix and consequently proliferate and differentiate therein. In the second phase, the construct is implanted in the appropriate anatomic location, where remodeling *in vivo* is intended to recapitulate the normal functional architecture of an organ or a tissue [611,612]. The key processes occurring during both *in vitro* and *in vivo* phases of tissue formation and maturation are: (1) cell proliferation, sorting and differentiation, (2) extracellular matrix production and organization, (3) biodegradation of the scaffold, (4) remodeling and potentially growth of the tissue.

To achieve the goal of tissue reconstruction, the scaffolds must meet several specific requirements [151,152,605]. A reasonable surface roughness is necessary to facilitate cell seeding and fixation [613,614]. A sufficient mechanical strength and stiffness are mandatory to oppose contraction forces and later for the remodeling of damaged tissues. A high porosity and adequate pore dimensions (Table 2 and Table 6) are very important to allow cell migration, vascularization, as well as diffusion of nutrients [352]. Namely, scaffolds should have a network of interconnected pores where more than ~60% of the pores should have a size ranging from ~150 μm to ~400 μm and at least ~20% should be smaller than ~20 μm [11,105,352,362,362,409,410,411,412,413,414,415,457,615,616,617,618,619,620,621]. Scaffolds must be manufactured from materials with controlled biodegradability and/or bioresorbability, such as calcium orthophosphate bioceramics, so that new bone will eventually replace the scaffold [622]. Furthermore, the resorption rate has to coincide as much as possible with the rate of bone formation (*i.e*., between a few months and about two years) [623]. This means that while cells are fabricating their own natural matrix structure around themselves, the scaffold is able to provide structural integrity within the body and eventually it will break down leaving the newly formed tissue that will take over the mechanical load. Besides, scaffolds should be easily fabricated into a variety of shapes and sizes [624] and be malleable to fit irregularly shaped defects. In many cases, ease of processability, such as an easiness of conformation, and injectability of calcium orthophosphate cements and concretes [149,463,464], can determine the choice of a certain biomaterial. Finally, sterilization with no loss of properties is a crucial step in scaffold production at both a laboratory and an industrial level [603]. In conclusion, since calcium and orthophosphate ions regulate bone metabolism, calcium orthophosphates appear to be among the few bone graft substitute materials that can be considered as a drug [1].

**Table 6:** A hierarchical pore size distribution that an ideal scaffold should exhibit [11].

Pore sizes of a 3D scaffold	Biochemical effect or function
<1 μm	Interaction with proteins
	Responsible for bioactivity
1–20 μm	Type of cells attracted
	Cellular development
	Orientation and directionality of cellular ingrowth
100–1000 μm	Cellular growth
	Bone ingrowth
	Predominant function in the mechanical strength
>1000 μm	Implant functionality
	Implant shape
	Implant esthetics

**Figure 13:**
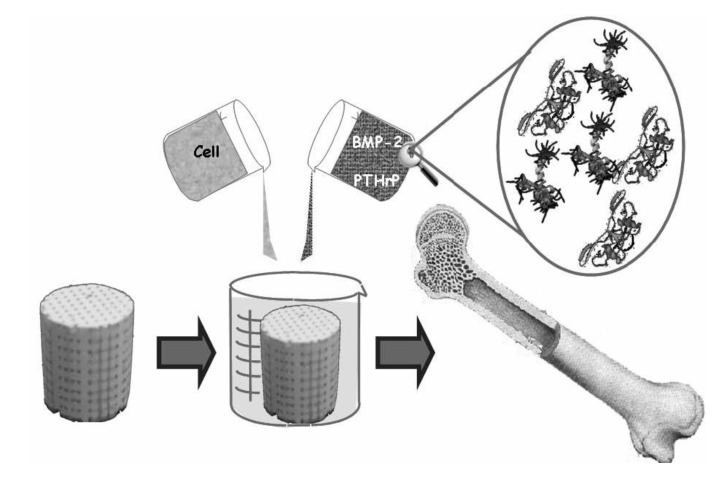
A schematic view of a third generation biomaterial, in which porous calcium orthophosphate bioceramic acts as a scaffold or template for cells, growth factors, *etc*. Reprinted from [42,52] with permission.

Many fabrication techniques are available to produce porous calcium orthophosphate scaffolds (Table 2) with varying architectural features (for details, see “Forming and shaping” and “Porosity” sections above). In order to achieve the desired properties at minimum expense, the production process should be optimized [625]. With the advent of tissue engineering, the search is on for the ultimate option—a “tissue engineered bone substitute”, consisting of a synthetic calcium orthophosphate scaffold impregnated with cells and growth factors. Figure 13 schematically depicts a possible fabrication process of such an item that, afterwards, will be implanted into a living organism to induce bone regeneration [42,52].

From the structural perspective, a degree of scaffold porosity is responsible for regulating the bioactivity of bone graft substitutes as a function of its influence on structural permeability, which controls the initial rate of bone regeneration and the local mechanical environment, which mediates the equilibrium volume of new bone within the repair site. Parameters such as pore interconnectivity, pore geometry, strut topography and strut porosity all contribute to modulate this process of osteogenesis and act synergistically to promote or screen the osteoconductive or osteoinductive potential of bone graft substitutes [412,626,627]. However, since bones have very different structures depending on their functions and locations, the same pore sizes and shapes may not be ideal for all potential uses. Therefore, bioceramic scaffolds of various porosities are required.

### 8.3. Scaffolds from Calcium Orthophosphate Bioceramics

Philosophically, the increase in life expectancy requires biological solutions to orthopedic problems previously managed with mechanical solutions. Therefore, since the end of 1990s, biomaterials research has focused on tissue regeneration instead of tissue replacement [628]. The alternatives include use of hierarchical bioactive scaffolds to engineer *in vitro* living cellular constructs for transplantation or use of bioresorbable bioactive particulates or porous networks to activate *in vivo* the mechanisms of tissue regeneration [629,630]. Thus, the aim of calcium orthophosphate bioceramics is to prepare artificial porous scaffolds able to provide the physical and chemical cues to guide cell seeding, differentiation and assembly into 3D tissues of a newly formed bone [587,631,632,633,634,635]. Particle sizes, shape and surface roughness of scaffolds are known to affect cellular adhesion, proliferation and phenotype. Additionally, the surface energy may play a role in attracting particular proteins to the bioceramic surface and, in turn, will affect the cells’ affinity to the material. More to the point, cells are exceedingly sensitive to chemical composition and their bone-forming functions can be dependent on grain morphology of the scaffolds. For example, osteoblast functions were found to increase on nanofiber structures if compared to nanospherical ones because nanofibers more closely approximate the shape of biological apatite in bones [636]. Besides, a significantly higher osteoblast proliferation on HA bioceramics sintered at 1200 °C as compared to that on HA bioceramics sintered at 800 °C and 1000 °C was reported [637]. Thus, to meet the tissue engineering requirements, much attention is devoted to further improvements of calcium orthophosphate bioceramics [638]. From the chemical point of view, the development includes synthesis of novel ion-substituted calcium orthophosphates [7,8,9,10,11,12,13,14,15,16,17,18,19,20,21,22,23,24,25,26,27,28,29,30,31,32,33,34,35,36,37]. From the material point of view, the major research topics include nanodimensional and nanocrystalline structures [639,640,641,642,643], organic-inorganic biocomposites and hybrid biomaterials [292], fibers, whiskers and filaments [644,645,646,647,648,649,650,651,652,653,654,655,656], micro- and nanospheres and beads [656,657,658,659,660,661,662,663,664,665,666,667,668,669], porous 3D scaffolds made of ACP [418], TCP [396,397], HA [167,364,365,398,400,441,625,670,671,672,673,674,675] and various biphasic formulations [440,667,676,677,678,679,680], structures with graded porosity [377,438,440,524,525,526,527,528,529] and hierarchically organized ones [681,682]. Furthermore, an addition of defects through an intensive milling [683,684] or their removal by a thermal treatment [685] can be used to modify a chemical reactivity of calcium orthophosphates. In addition, more attention should be paid to crystallographically aligned calcium orthophosphate bioceramics [686].

There are three principal therapeutic strategies for treating diseased or injured tissues in patients: (i) implantation of freshly isolated or cultured cells; (ii) implantation of tissues assembled *in vitro* from cells and scaffolds; (iii) *in situ* tissue regeneration. For cellular implantation, individual cells or small cellular aggregates from the patient or a donor are either injected into the damaged tissue directly or are combined with a degradable scaffold *in vitro* and then implanted. For tissue implantation, a complete 3D tissue is grown *in vitro* using patient or donor cells and a bioresorbable scaffold and then is implanted into the patients to replace diseased or damaged tissues. For *in situ* regeneration, a scaffold implanted directly into the injured tissue stimulates the body’s own cells to promote local tissue repair [595,687]. In any case, simply trapping cells at the particular point on a surface is not enough: the cells must be encouraged to differentiate, which is impossible without the presence of suitable biochemical factors [688]. All the previously mentioned clearly indicate that for the purposes of tissue engineering, calcium orthophosphate bioceramics play an auxiliary role; namely, they act as a suitable material to manufacture the appropriate 3D templates, substrates or scaffolds to be colonized by living cells before the successive implantation. The *in vitro* evaluation of potential calcium orthophosphate scaffolds for tissue engineering has been described elsewhere [689], and data on the mechanical properties of calcium orthophosphate bioceramics for use in tissue engineering are also available [690,691]. The effect of a HA-based biomaterial on gene expression in osteoblast-like cells was reported as well [692]. To conclude, the excellent biocompatibility of calcium orthophosphate bioceramics, their possible osteoinductivity [168,415,457,551,552,553,554,555,556,557,558,559,560,561,562,563] and a high affinity for drugs, proteins and cells make them very functional for tissue engineering applications. The feasible production of scaffolds with tailored structures and properties opens up a spectacular future for calcium orthophosphates [692,693,694,695,696,697,698,699].

### 8.4. A Clinical Experience

During the last decade, several groups have made steps towards a clinical application of cell-seeded calcium orthophosphate bioceramics for bone tissue engineering of humans. For example, Quarto *et al.* [700] were the first to report treatment of large (4–7 cm) bone defects of the tibia, ulna and humerus in three patients from 16 to 41 years old, where conventional surgical therapies had failed. The authors implanted a custom-made unresorbable porous HA scaffold seeded with *in vitro* expanded autologous bone marrow stromal cells. In all three patients, radiographs and computed tomographic scans revealed abundant callus formation along the implants and good integration at the interfaces with the host bones by the second month after surgery [700]. In the same year, Vacanti *et al.* [701] reported the case of a man who had a traumatic avulsion of the distal phalanx of a thumb. The phalanx was replaced with a specially treated natural coral (porous HA; 500-pore ProOsteon (see Table 3)) implant that was previously seeded with *in vitro* expanded autologous periosteal cells. The procedure resulted in the functional restoration of a stable and biomechanically sound thumb of normal length, without the pain and complications that are usually associated with harvesting a bone graft.

Morishita *et al.* [702] treated a defect resulting from surgery of benign bone tumors in three patients using HA scaffolds seeded with *in vitro* expanded autologous bone marrow stromal cells after osteogenic differentiation of the cells. Two bone defects in a tibia and one defect in a femur were treated. Although ectopic implants in nude mice showed the osteogenicity of the cells, details such as the percentage of the implants containing bone and at what quantities were not reported. Furthermore, cell-seeded calcium orthophosphate scaffolds were found to be superior to autograft, allograft or cell-seeded allograft in terms of bone formation at ectopic implantation sites [703].

To conclude this part, one should mention that a cell seeding method for practical clinical experience is not enough. Although cell seeding into scaffolds at high density is closely associated with enhancement of tissue formation in 3D constructs (*i.e.*, cartilage [704] and bone [705]), effective and high-density cell seeding into 3D scaffolds is difficult to achieve. Technical difficulties in cell seeding are caused by the complex structure of the scaffold and insufficient migration into the scaffolds due to pore size and material, which prolongs the culture period because of the shortage of initially seeded cells. Recently, Shimizu *et al.* [706] proposed a methodology for tissue engineering using magnetite nanoparticles and magnetic force. This method has been applied to a cell seeding process and attractive results have been reported.

## 9. Conclusions and Outlook

The available chronology of the search for a suitable bioceramic for bone substitutes is as follows: since the 1950s, the first aim was to use bioinert [533] bioceramics, which had no reaction with living tissues. Later on, in the 1980s, the trend changed towards the opposite: the idea was to implant bioceramics that reacted with the surrounding tissues by producing newly formed bone. These two stages have been referred to as the first and the second generations of bioceramics, respectively [707]. In the current century, we search for the third generation of bioceramics [687], which will be able to regenerate bone tissues by stimulating specific responses at the molecular level [42,52]. One should note that these three generations should not be interpreted as the chronological but the conceptual, since each generation represents an evolution on the requirements and properties of the biomaterials involved. This means that at present, research and development is still devoted to biomaterials and bioceramics that, according to their properties, could be considered to be of the first or the second generations, because the second generation of bioceramics with added porosity is one of the initial approaches in developing the third generation of bioceramics [708]. Furthermore, there is another classification of the history of biomaterials introduced by James M. Anderson. According to Anderson, between 1950–1975, researchers studied bio*materials*, between 1975–2000 they studied *biomaterials* and since 2000 the time for *bio*materials has been coming [709]; here, the italicized letters emphasize the major direction of the research efforts in the complex subject of biomaterials. As bioceramics are biomaterials of the ceramic origin (see Section 2), Anderson’s historical classification appears to be applicable to the bioceramics field.

The field of biomaterials is in the midst of a revolutionary change in which the life sciences are becoming equal in importance to materials science and engineering as the foundation of the field. Simultaneously, advances in engineering (for example nanotechnology) are greatly increasing the sophistication with which biomaterials can be designed, allowing fabrication of biomaterials with increasingly complex functions [76]. Specifically, during the last ~40 years, calcium orthophosphate bioceramics has become an integral and vital segment of our modern health care delivery system. In the modern fields of the third generation bioceramics (Hench) or BIOceramics (Anderson), the full potential of calcium orthophosphates has only begun to be recognized. Namely, calcium orthophosphates, which were intended as osteoconductive bioceramics in the past, stand for materials to fabricate osteoinductive implants nowadays [168,415,457,551,552,553,554,555,556,557,558,559,560,561,562,563]. The initial steps in this direction have been already made by both fabricating BCP-based scaffolds for bone tissue engineering through the design of controlled 3D-porous structures and increasing the biological activity through development of novel ion-substituted calcium orthophosphate bioceramics [11,417]. In the future, the composition, microstructure and molecular surface chemistry of various types of calcium orthophosphates will be tailored to match the specific biological and metabolic requirements of tissues or disease states. This new generation of calcium orthophosphate bioceramics should enhance the quality of life of millions of people as they grow older [710].

In spite of the great progress, there is still great potential for major advances to be made in the field of calcium orthophosphate bioceramics [4]. This includes requirements for:

- Improvement of the mechanical performance of existing types of bioceramics;
- Enhanced bioactivity in terms of gene activation;
- Improvement in the performance of biomedical coatings in terms of their mechanical stability and ability to deliver biological agents;
- Development of smart biomaterials capable of combining sensing with bioactivity;
- Development of improved biomimetic composites.

Furthermore, there is still need for a better understanding of the biological systems. For example, the bonding mechanism between the bone mineral and collagen remains unclear. It is also unclear whether a rapid repair that is elicited by the new generation of bioceramics results from the enhancement of mineralization *per se* or whether there is a more complex signaling process involving proteins in collagen. If we were able to understand the fundamentals of bone response to specific ions and the signals they activate, then we could design better bioceramics for the future [4].

To finalize this review, it is obvious that the present status of research and development in the field of calcium orthophosphate bioceramics is still at the starting point for the solution of new problems at the confluence of materials science, biology and medicine, concerned with the restoration of damaged functions in humans. A large increase in active elderly people has dramatically raised the need for load-bearing bone graft substitutes, for example for bone reconstruction during revision arthroplasty or for the reinforcement of osteoporotic bones. Strategies applied in the last four decades towards this goal have failed. New strategies, possibly based on self-assembling and/or nanofabrication, have to be proposed and developed [711]. Furthermore, it should be feasible to design a new generation of gene-activating calcium orthophosphate based scaffolds tailored for specific patients and disease states in the future. Perhaps, bioactive stimuli will be used to activate genes in a preventative treatment to maintain the health of aging tissues. Currently this concept seems impossible. However, we need to remember that only ~40 years ago the concept of a material that would not be rejected by living tissues also seemed impossible [542].
